# Identification of candidate genes involved in Zika virus-induced reversible paralysis of mice

**DOI:** 10.1038/s41598-025-86475-0

**Published:** 2025-01-23

**Authors:** John D. Morrey, Venkatraman Siddharthan, Hong Wang, Alexandre L. R. Oliveira, Keiichiro Susuki, Rakesh Kaundal, Sara M. Freeman, Aaron J. Thomas, Naveen Duhan, Nathan G. Corry

**Affiliations:** 1 Institute for Antiviral Research, Department of Animal, Dairy, and Veterinary Sciences, Utah State University, Logan, UT 84321-5600 USA; 2 Institute of Biology, University of Campinas, Campinas, SP Brazil; 3 Department of Neuroscience, Cell Biology and Physiology, Boonshoft School of Medicine, Wright State University, Dayton, OH 45435-0001 USA; 4 Bioinformatics Facility, Center for Integrated BioSystems, Department of Plants, Soils, and Climate, College of Agriculture and Applied Sciences, Logan, UT 84322 USA; 5 Department of Computer Science, College of Science, Logan, UT 84322 USA; 6 Department of Biology, Utah State University, Logan, UT 84322 USA; 7 Department of Animal, Dairy, and Veterinary Sciences, Utah State University, Logan, UT 84322 USA; 8 Department of Plants, Soils, and Climate, College of Agriculture and Applied Sciences, Logan, UT 84322 USA

**Keywords:** Microbiology, Virology, Viral pathogenesis, Neurology, Neurological disorders, Central nervous system infections

## Abstract

Zika virus (ZIKV) causes a variety of peripheral and central nervous system complications leading to neurological symptoms such as limb weakness. We used a mouse model to identify candidate genes potentially involved in causation or recovery from ZIKV-induced acute flaccid paralysis. Using *Zikv* and *Chat* chromogenic and fluorescence in situ RNA hybridization, electron microscopy, immunohistochemistry, and ZIKV RT-qPCR, we determined that some paralyzed mice had infected motor neurons, but motor neurons are not reduced in number and the infection was not present in all paralyzed mice; hence infection of motor neurons were not strongly correlated with paralysis. Consequently, paralysis was probably caused by by-stander effects. To address this, we performed bioinformatics analysis on spinal cord RNA to identify 2058 differentially expressed genes (DEGs) that were altered during paralysis and then normalized after paralysis. Of these “biphasic” DEGs, 951 were up-regulated and 1107 were down-regulated during paralysis, followed by recovery. To refine the search for candidate DEGs we used gene ontology analysis and RT-qPCR to select 3 DEGs that could be involved with the node of Ranvier function and 5 DEGs that could be involved with synaptic function. Among these, *SparcL1:Sparc* DEG ratios were identified to be inversely correlated with ZIKV-induced paralysis, which is consistent with the known function of SPARC protein to antagonize the synaptogenesis of SPARCL1. *Ank3*, *Sptbn1*, and *Epb41l3* affecting the structures at and near the nodes of Ranvier were significantly downregulated during ZIKV-induced paralysis. The primary contribution is the identification of 8 candidate genes that may be involved in the causation or recovery of ZIKV-induced paralysis.

## Introduction

A variety of neurological complications have been described in patients with Zika virus (ZIKV) infection. Congenital ZIKV syndrome and Guillain-Barré syndrome (GBS) are two serious outcomes associated with ZIKV outbreaks^1–4^. In addition to GBS, ZIKV infection can trigger central nervous system disorders such as acute disseminated encephalomyelitis, transverse myelitis, and encephalitis^5,6^. The World Health Organization declared ZIKV as a cause of GBS based on epidemiological evidence^7^ and has been further confirmed with subsequent epidemiological studies^2^. GBS is an acute peripheral neuropathy diagnostically indicative of an autoimmune disease characterized by limb weakness^8,9^. Fifty to seventy-five percent of GBS cases occur within a couple of weeks after a respiratory or gastrointestinal infection or perhaps other immune stimuli that triggers immune responses affecting the peripheral nerves and spinal roots^10^. Even though GBS is a self-limiting disease, the patients with ZIKV-associated GBS show considerable long-term disability since treatment options are very limited^11^. To manage ZIKV-associated neurological conditions, a more detailed knowledge of pathophysiology is required.

To investigate the pathophysiology, we used the only mouse model of reversible ZIKV-induced acute flaccid paralysis^12^. Fifteen to twenty percent of aged interferon αβ-receptor knockout (IFNAR^−/−^) mice develop acute hindlimb flaccid paralysis 8–11 days after ZIKV challenge. They recover within a week. Motor neurons are not destroyed despite infection of the lumbar spinal cord with the virus. Characteristic feature of the electrophysiological studies in this mouse model was the isolated F-wave abnormalities^12^. The F-wave occurs from the backfiring of antidromically activated ventral horn neurons. Its measurement helps in assessing motor conduction along the entire length of the peripheral axons, including the most proximal segment. F-waves could readily be detected in hindlimbs of sham-infected mice; but they were absent in 3 of 4 hindlimbs of paralyzed mice, whereas M-waves were preserved indicating the distal motor axons were intact^12^. F-waves could be detected in ZIKV-infected mice without overt paralysis, but their F-wave latencies were much greater than control mice. Similarly, absence of F-waves with preserved M-waves has been reported in GBS with anti-ganglioside antibodies^13^. These anti-ganglioside antibodies cause autoimmune attack at the nodes of Ranvier in ventral roots^14^, and the nodal disruption is the early pathological feature of the axonal form of GBS^15,16^. Therefore, we reasoned that nodal disruption may contribute to the pathophysiology of acute flaccid paralysis associated with ZIKV infection.

Alternatively, isolated F-wave abnormalities may be due to disrupted synaptic transmission in spinal motor neurons. A recent study suggested that F-waves are mediated by spinal microcircuits activated by recurrent motor axon collaterals via glutamatergic synapses^17^. Spinal cord α-motor neurons, the largest motor neurons, innervate extrafusal muscle fibers to provide the most force during muscle contraction^18^. They receive synaptic inputs from different sources including descending pathways of the corticospinal tract, and primary sensory neurons located in the dorsal root ganglia. Every α-motor neuron receives about 100,000 inputs, when considering the cell body and dendritic tree^19^. Normally, ~ 50% of the cell body is covered with presynaptic terminals in rodents^20^. Morphology by EM is used to identify types of synaptic boutons. C-boutons identify α-motor neurons, F-boutons (flattened vesicle) provide inhibitory signals, and S-boutons (spherical vesicle) provide excitatory signals^12^. Following injury or during the course of neurodegenerative diseases, such as in the experimental autoimmune encephalomyelitis (EAE) temporary paralysis model^21^, the covering of presynaptic terminals changes with loss of excitatory boutons, together with a decrease in the clustering of terminals, which has been correlated with transient paralysis^21^. During paralysis of ZIKV-infected IFNAR^−/−^ mice, the percent coverage of α-motor neurons by boutons was reduced by 20%. Moreover, S- and F-boutons were lowered during paralysis, but were normalized in recovering mice^12^.

In this study, we performed bioinformatics analyses of next-generation RNA sequencing (RNA seq) data to identify differentially expressed genes (DEGs) of paralyzed mice compared to both sham-infected mice and mice recovering from paralysis, which are refer to in the text as “biphasic” DEGs. We reasoned that gene expression altered during paralysis, and then normalized during recovery would be more likely to code for affecter proteins of paralysis and then recovery. We filtered for biphasic DEGs associated with non-cytolytic α-motor neuron pathologies including synaptic retraction of pre- and post-synaptic terminals, and for biphasic DEGs associated with nodes of Ranvier.

## Results

### Zikv and choline acetyltransferase (CHAT) chromogenic in situ RNA hybridization

**Fig. 1:**
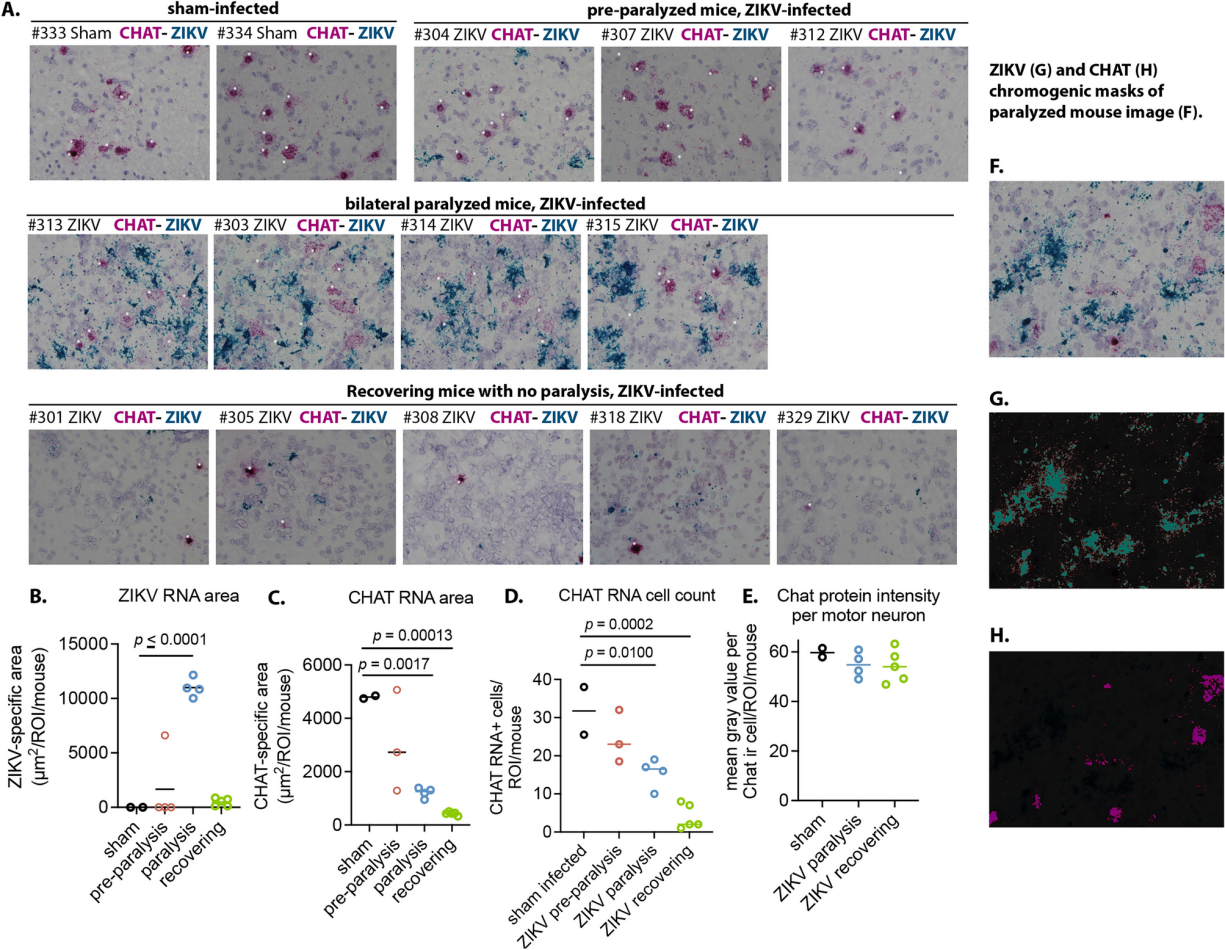
Increased *Zikv* RNA during paralysis and reduced *Chat* RNA in lumbosacral spinal cords during and after paralysis of ZIKV-infected IFNAR^−/−^ mice (Study design #1). (**A**) In situ hybridization using RNAscope™ from paralyzed (#313, #303, #314, #315), recovering (#301, #305, #308, #318, #329), and pre-paralyzed (#304, #307, #312) IFNAR^−/−^ mice infected with ZIKV and from sham-infected mice (#333, #334). *Chat* channel 1 (red) and *Zikv* channel 2 (blue). White asterisks indicate *Chat* RNA + cells. (**B**) *Zikv* RNA-specific area during paralysis predominently in the region of interest (ROI) in the vicinity of lamina IX of the ventrolateral cord containing α-motor neurons. (**C**) Declining *Chat* RNA-specific area. (**D**) Declining number of *Chat* RNA + neurons. (**E**) CHAT protein immunoreactivity (ir) remained constant. *Zikv* (**G**) and *Chat* (**H**) chromogenic masks of #303 paralyzed mouse (**F**) for quantitative analysis using BZ-X800 Analyzer software (Keyence Corporation, v 1.1.2.4). Statistics: One-way ANOVA with Dunnett’s multiple comparison test.

CHAT was used as a definitive marker for cholinergic neurons^22^ and more specifically as a marker for motor neurons in the ventral horn of the spinal cord^23^. The previous publication describing this model^12^ did not identify colocalization of ZIKV immunoreactivity (ir) with CHAT ir (marker for motor neurons), suggesting that the motor neurons do not die because they are not infected. In this report (Study design #1, “Methods”), we investigated colocalization of *Zikv* RNA with *Chat* RNA using chromographic in situ hybridization (Fig. 1). The white asterisks in the images identify *Chat* RNA-positive cells. The lack of colocalization of the *Chat* RNA-positive cells with the blue *Zikv* RNA suggests that the virus did not infect the motor neurons despite infection of the spinal cord. As previously observed^12^, motor neurons were not killed during paralysis as observed with unchanged CHAT protein ir (Fig. 1E). However, *Chat* RNA area (Fig. 1C) and cell counts (Fig. 1D) steadily decreased in pre-paralysis, paralysis, and recovering mice, but the CHAT protein immunoreactivity was stable.

### Zikv and chat fluorescence in situ RNA hybridization

**Fig. 2:**
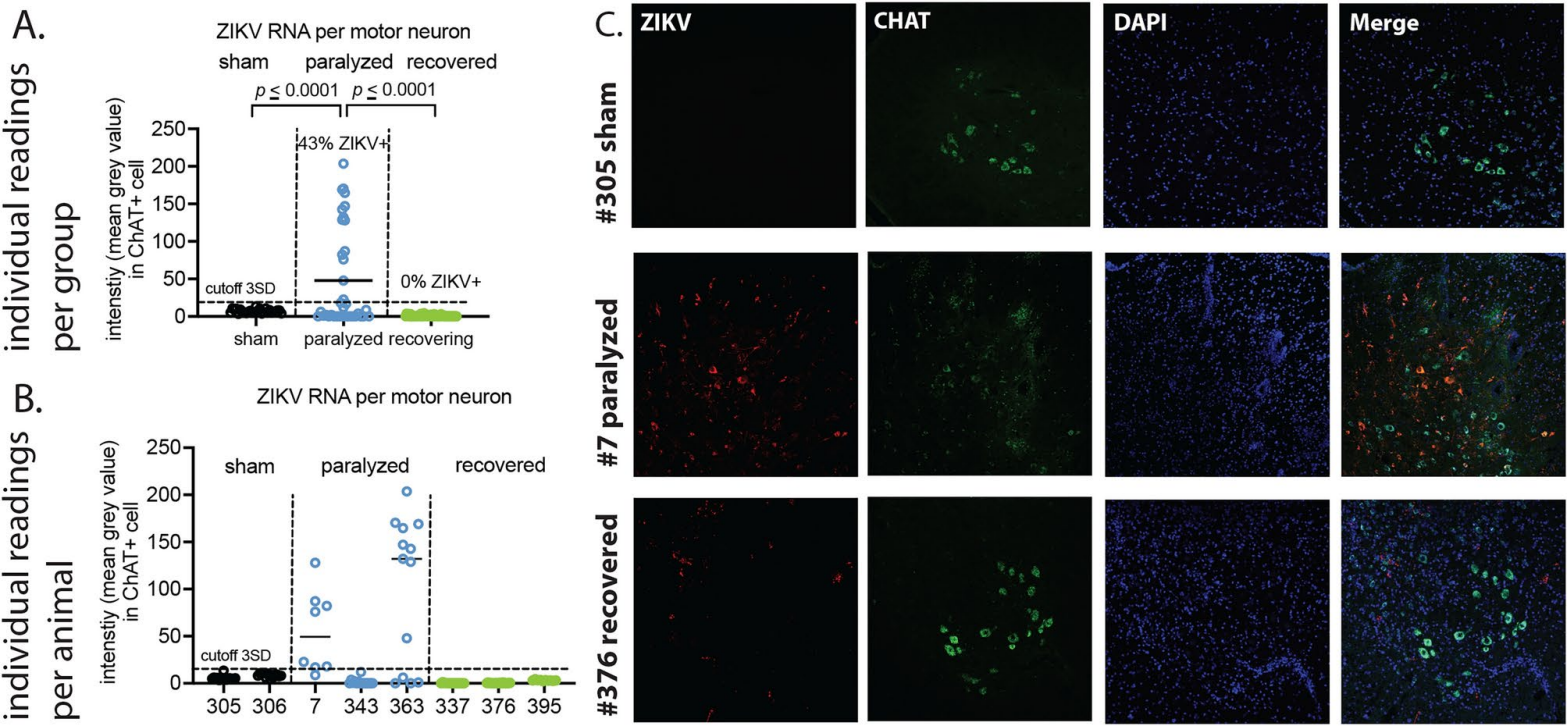
*Zikv* RNA intensities of fluorescence of in situ RNA hybridization co-localized with *Chat* RNA-positive motor neurons in sham, paralyzed and recovering IFNAR^−/−^ mice (5.3 months old) infected with ZIKV. (**A**) Individual readings per group. (**B**) Individual readings per animal. (**C**) Example images of *Zikv* and *Chat* RNA -positive cells of sham-infected mouse #305, infected paralyzed mouse #7, and recovering mouse #376 in the vicinity of lamina IX of the ventrolateral cord. Spinal cords of recovering mice were obtained within 2 to 3 days after cessation of paralysis on days 9 to 13 after viral challenge.

To increase the sensitivity of possible *Zikv* RNA co-localized with *Chat* RNA-positive cells, fluorescence in situ RNA hybridization was use (Fig. 2C) (Study design #2). Forty-three percent (16/37) of *Chat* RNA-positive cells were co-localized with *Zikv* RNA (Fig. 2A). *Chat* RNA-positive cells of two of three paralyzed mice (#7, #363) were statistically positive (above mean + 3SD) for *Zikv* RNA (Fig. 2B, left), but the other paralyzed mouse (#343) did not have detectable *Zikv* RNA. None of the motor neurons from 3 recovering mice had detectable *Zikv* RNA colocalized with *Chat* RNA.

### Electron microscopy

**Fig. 3:**
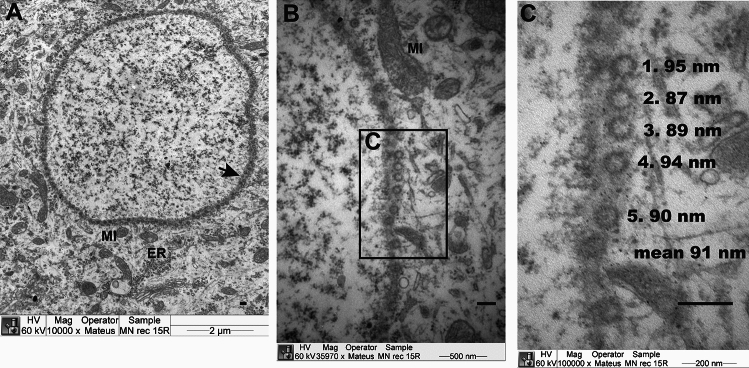
Novel structure in motor neurons of paralyzed mice not associated with Zika virus particles from tissued of a prior study^12^. Two motor neurons with synaptic retraction from a ZIKV-induced paralyzed IFNAR^−/−^ mouse were examined by electron microscopy for the presence of any immature ZIKV particles of ~ 60 nm^24^ and mature particles with a diameter of ~ 50 nm with an electron dense core of 30 nm diameter. The electron microscopy method is described^12^. Two motor neurons from a sham-infected mouse were also examined. (**A**) Novel structure (NS) mitochondria (MI), endoplasmic reticulum (ER), and an array of particles or vesicles (arrow). (**B**) Magnified (35,970×) section of array, (**C**) Diameter of particles or vesicles in magnified array (100,000×). The mean of two x–y diameter measurements for each of 5 particles at each of 5 different locations are shown. Overall average = 93.5 nm, n = 25. Scale bars (200 nm) and legends shown in images.

Two motor neurons with synaptic retraction from a ZIKV-induced paralyzed IFNAR^−/−^ mouse and from a mouse with paresis from the prior publication^12^ were examined for immature ZIKV particles of ~ 60 nm^24^ or mature particles with a diameter of ~ 50 nm with an electron dense core of 30 nm diameter. Novel structures of infected cells included particles or vesicles with 87 to 95 nm diameters (Fig. 3), but they were not characteristic of ZIKV particles. Previously observed sheet-like structures in ZIKV-infected cell culture^25^ were not observed in the 3 motor neurons from mice with motor deficits of this study. ZIKV-specific particles were not observed in these motor neurons. These data suggest that some α-motor neurons from paralyzed mice were not infected with ZIKV.

### Immunohistochemical analysis (IHC)

**Fig. 4:**
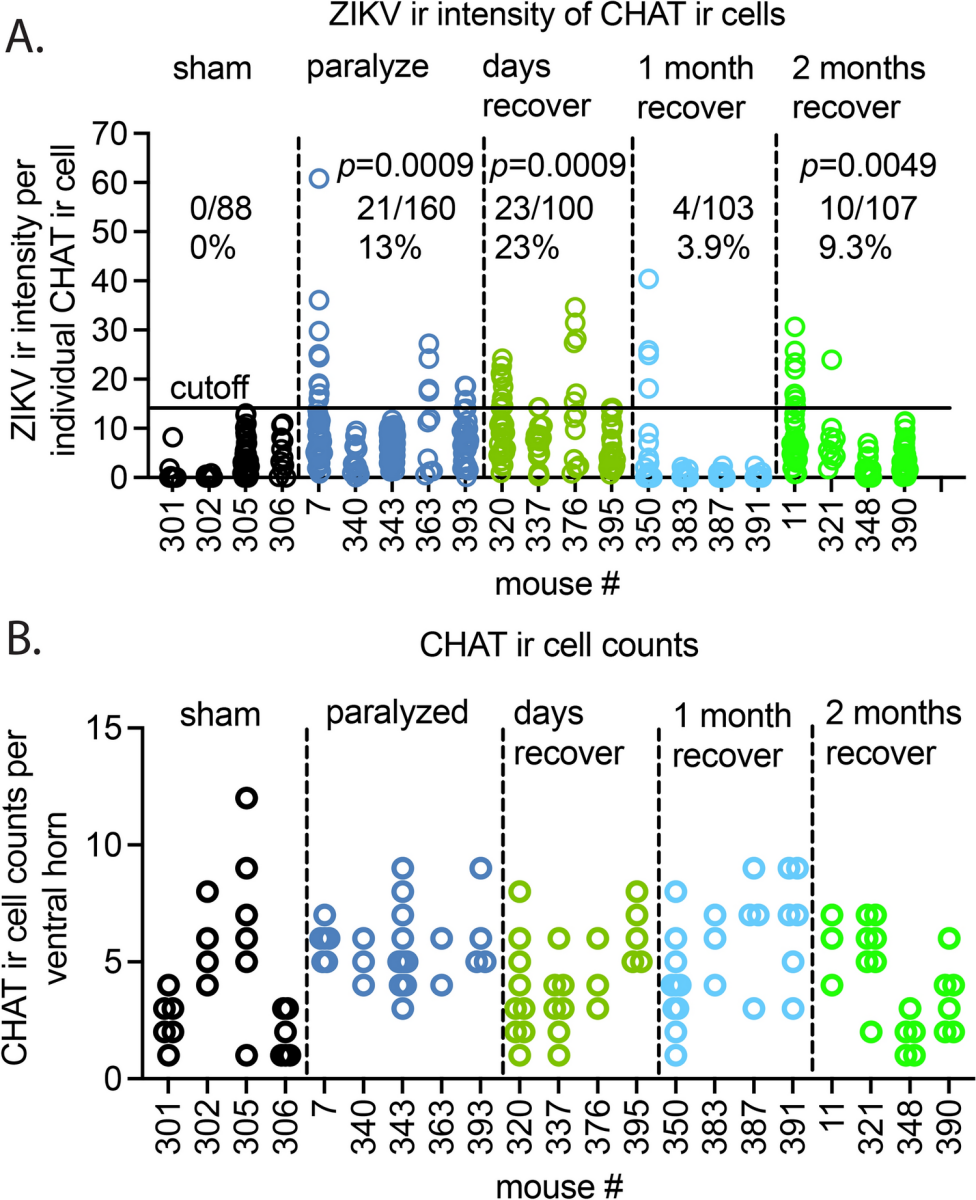
ZIKV infection of α-motor neurons (large CHAT ir cells) through 2 months after recovery from paralysis (Study design #2). (**A**) ZIKV ir intensity per individual CHAT ir cells. The cutoff line was 3SD above the mean of individual measurements (n = 88) of sham-infected mice (solid horizontal line). Measurements above the cutoff were considered positive/total measurements (ratios and percentage). No statistical significance was identified when the numbers of positive mice were compared with 0 of 4 negative sham-infected mice. Statistics: Chi-square analysis. (**B**) CHAT ir cell counts per ventral horn slice.

IHC analysis of ZIKV and CHAT proteins was also performed on lumbosacral spinal cords of these same mice and more mice at 1 and 2 months after recovery from paralysis (Fig. 4) (Study design #2). Three standard deviations of the mean of ZIKV ir intensities per CHAT ir positive cells from 4 sham-infected mice (Fig. 4A, solid horizontal line) were considered the cutoff for negative ZIKV infection. According to this criteria, 21/160 Sects. (13%) of Chat ir-positive motor neurons from paralyzed mice were considered positive for ZIKV antigen. Not all paralyzed mice (2/5) had detectable ZIKV ir (#340, #343). Even though *Zikv* RNA was not detected in recovering mice by fluorescence in situ hybridization (Fig. 2), ZIKV antigen ir was still detected in 23%, 3.9%, and 9.3% of mice that had recovered from paralysis within days, 1 month, and 2 months, respectively. These data and *Zikv* RNA data (Fig. 2) suggested that viral RNA replication ceased within days after recovery, but the ZIKV protein persisted in some animals out to 2 months after paralysis.

**Fig. 5:**
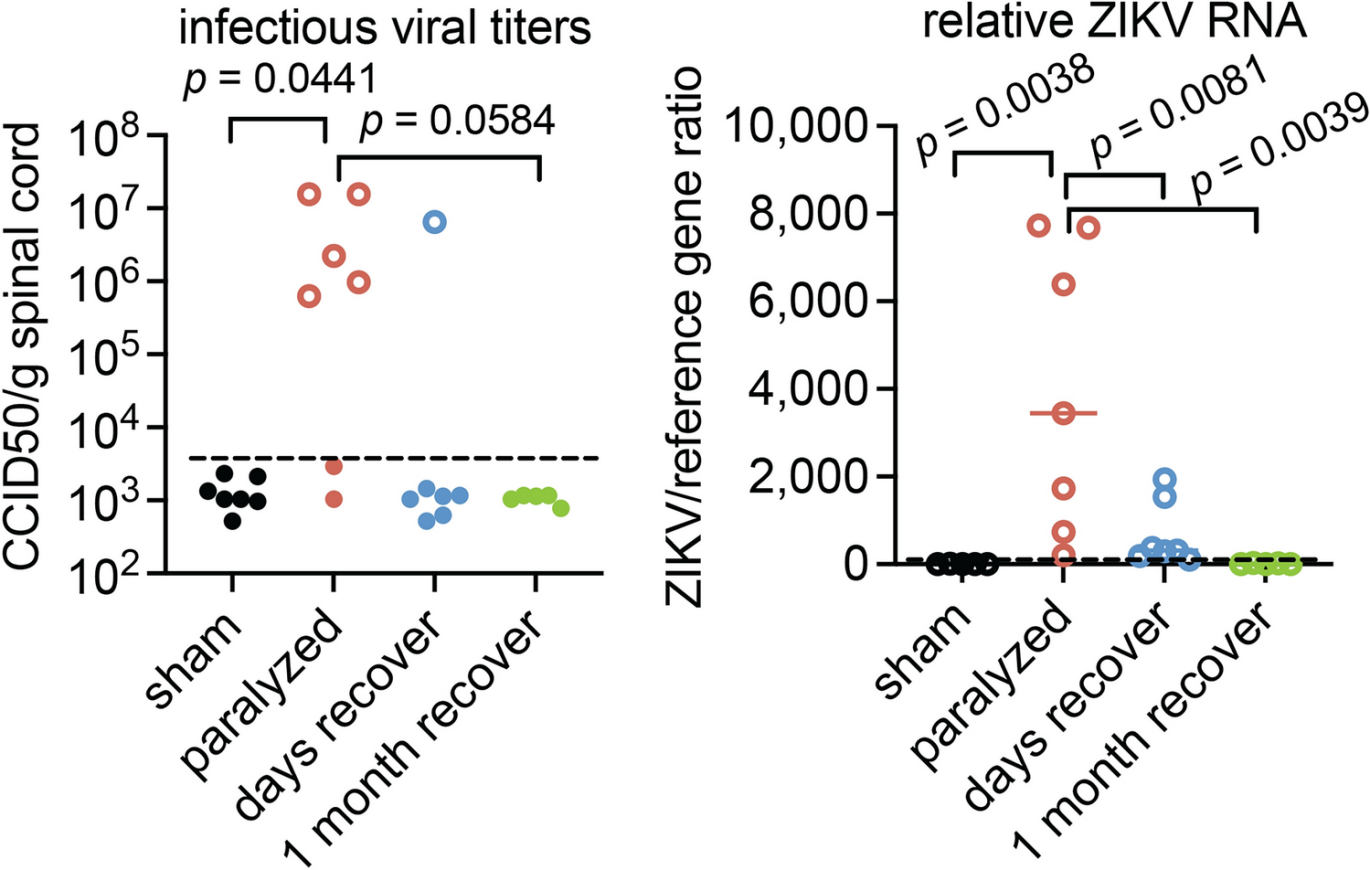
Infectious cell culture titers and *Zikv* RNA (RT-qPCR) were performed on spinal cord homogenates (Study #3). Open circles are samples with quantifiable viral titers. Solid circles are samples confirmed to be negative by serially passaging the spinal cord homogenates 2 times on indicator cells (Vero 76) and then quantifying the supernatant. Dotted lines are 3 standard deviations of the mean. Statistics: Chi-square analysis.

We performed infectious viral titer and *Zikv* RNA (RT-qPCR) assays on lumbosacral cords collected from paralyzed mice and mice that had recovered from paralysis within days, or 1 month after paralysis (Study design #3). As expected, no infectious virus or viral RNA was detected in sham-infected mice, whereas infectious virus was detected in spinal cords of paralyzed mice except for two mice that had no quantifiable or amplified infectious virus in serially passaged indicator cells (Fig. 5, solid circles). All but one of the samples from mice having recovered from visible paralysis within 2 days were confirmed to have no infectious virus. None of the mice at 1-month post-paralysis had infectious virus. The spinal cords from paralyzed mice with no detectable infectious virus (Fig. 5), along with no detection of viral particles by EM (Fig. 3) and no detectable in situ* Zikv* RNA in motor neurons of one of three paralyzed mice (Fig. 2B), suggested that infectious virus in ChAT positive motor neurons was not necessary to cause paralysis.

### Bioinformatics

We used bioinformatics analysis to search for candidate genes involved in the cause or recovery from ZIKV-induced paralysis. Spinal cords (Study design #2) were processed to enrich for motor neurons. Of the 27,489 genes covered by RNA seq, 9619 DEGs were identified between spinal cords of sham- compared to ZIKV-infected mice, and 2663 DEGs were identified between paralyzed mice and those mice having recovered within 2 to 3 days after paralysis (Table 1). Of those DEGs, we identified biphasic DEGs that were altered during paralysis and then normalized after paralysis. The numbers of up- and down-regulated biphasic DEGs were 951 and 1107, respectively, for a total of 2058 biphasic DEGs, which filtered down the numbers of candidate DEGs to 7% of the total.

**Table 1:** Number of up- and down-regulated biphasic DEGs.

Comparison groups	Total genes	Total DEGs^a^	Up-regulated biphasic DEGs^b^	Down-regulated biphasic DEGs^b^	Total biphasic DEGs
Sham/paralyzed^c^		9619			
	27,489		951	1107	2058
Paralyze/recovering^d^		2663			

^a^Total DEGs include non-biphasic and biphasic DEGs.

^b^The up- and down-regulated biphasic DEGs during paralysis were selected based on genes that change during paralysis and started to normalize during recovery. The sham-to-paralysis DEG comparisons had FDR ≤ 0.05, while the paralysis-to-recovering DEG comparisons may have had greater FDR values since the recovery may have been incomplete at the beginning stages.

^c^Comparing sham-infected mice to ZIKV-infected paralyzed (VPS = 5 or 6) mice.

^d^Comparing ZIKV-paralyzed mice with mice having recovered between 2–3 days after paralysis.

**Fig. 6:**
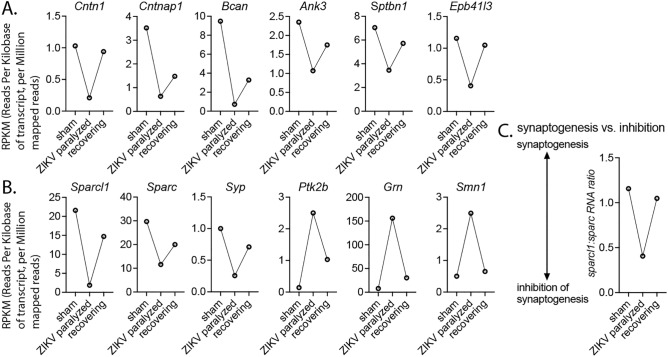
Biphasic DEG expression of genes known to affect the structures and functions of the (**A**) nodes of Ranvier and (**B**) synaptic retraction coincident with paralysis followed by recovery. (**C**) synaptogenesis vs. inhibition (*Sparcl1* vs. *Sparc*).* Ank3*, Ankyrin*. Bcan*, Brevican*. Cntn1*, Contactin 1*. Cntnap1*, Contactin associated protein-like 1*. Epb41l3*, spectrin beta, non-erythrocytic 1. *Grn*, Granulin. *Ptk2b*, Protein kinase 2. *Smn1*, Survival motor neuron 1. *Sparc*, Secreted acidic cysteine rich glycoprotein*. Sparcl1*, *Sparc-like 1. Syp*, synaptophysin*.*

In the spinal cords from paralyzed mice, we identified 10 biphasic DEGs in a total of 20 genes (50%) in nodes of Ranvier GO:0033268 term, 15/29 (52%) in axon initial segment GO:0043194 term, 5/12 (42%) in juxtaparanode GO:0044224 term, and 7/16 (44%) in the paranode GO:0033270 term. Based on biological relevance, FDR values, and up- or down-regulation during paralysis, we selected 6 DEGs for further analysis (Table 2): neuronal Ank3 at nodes^26^ and axon initial segment^27^, oligodendrocyte Ank3 at paranodes^28^; Bcan in the oligodendrocyte, axonal initial segment (AIS) and nodal extracellular matrix^29^; neuronal Cntn1^30^, Cntnap1^31^, and *Epb41l3*^32^ at paranodes; and Sptbn1 at paranodes^33,34^ annotated with the juxtaparanode and paranode GO terms (Fig. 6A).

**Table 2:** Candidate genes involved in synaptic retraction and nodes of Ranvier.

Biphasic DEG	Gene	Function	FDR values^b^	Up- or down-regulated
*Sparcl1*	Sparc like 1	Synapse^a^	7.2E−12 & 3.7E−03	Down
*Sparc*	Secreted protein acidic and cysteine rich	Synapse	1.1E−05 & 1.6E−01	Down
*Ptk2b*	Protein tyrosine kinase 2 beta	Synapse	3.8E−29 & 2.7E−03	Up
*Grn*	Granulin	Synapse	4.5E−18 & 3.0E−06	Up
*Syp*	Synaptophysin	Synapse	3.9E−05 & 2.6E−01	Down
*Smn1*	Survival of motor neuron 1	Synapse	1.9E−07 & 2.6E−08	Up
*Cntn1*	Contactin	Node^c^	1.0E−03 & 4.7E−02	Down
*Cntnap1*	Contactin Associated Protein-like 1	Node	1.6E−08 & 7.2E−02	Down
*Bcan*	Brevican	Node	2.9E−05 & 5.9E−02	Down
*Ank3 (AnkG)*	AnkyrinG	Node	2.4E−06 & 1.3E−01	Down
*Sptbn1*	Spectrin beta, non-ethrythocytic	Node	4.4E−05 & 1.4E−01	Down
*Epb41l3*	Erythrocyte membrane protein band 4.1 like 3	Node	4.6E−03 & 1.3E−01	Down

^a^Involved in synaptic function or structure.

^b^FDR (false discovery rate) value of sham-infected vs ZIKV-infected paralyzed mice & FDR value for paralyzed mice vs recovering mice within 2 to 3 days of paralysis.

^c^Involved in structure or function of nodes of Ranvier.

SynGO consortium was used to identify and organize GO terms related to synapse biology^35^. A list of compelling biphasic DEGs that could affect the function of synapses are shown (Table 2, Fig. 6B). *SparcL1* was identified to be a biphasic DEG (FDR = 7.2 × 10^–12^ during paralysis, FDR = 0.0037 during recovery) and was found by SynGO in the extracellular matrix of synaptic cleft GO:0098965 term and synaptic membrane adhesion GO:0099560 term. *Sparc* was also identified to be a biphasic DEG (FDR = 1.1 × 10^–5^ during paralysis, FDR = 0.16 during recovery) and was found by SynGO in regulation of gene organization GO:0050807 term and synapse GO:0045202 term. Sparcl1 increases synaptogenesis and Sparc inhibits Sparcl1 to inhibit synaptogenesis; therefore, a high value of the ratio of Sparcl1:Sparc^36,37^ likely favors synaptogenesis, and conversely, a low ratio-value favors inhibition of synaptogenesis. Since *SparcL1* gene expression was identified to be downregulated during ZIKV-induced paralysis in a biphasic manner, we calculated the *SparcL1:Sparc* ratios. As would be expected, the ratios were inversely correlated with ZIKV-induced paralysis followed by normalization during recovery (Fig. 6C), i.e., low ratios would favor inhibition of synaptogenesis during paralysis. *Syp* was also found to be downregulated during paralysis (Table 2). *Ptk2b*, *Grn*, and *Smn1* were upregulated.

### Validating candidate biphasic DEGs

To validate the bioinformatic data of putative biphasic DEGs, RT-qPCR was performed. To also verify consistency of biphasic DEG results between two different experiments, this RT-qPCR analysis was performed in a separate experiment (Study design #3) from the bioinformatics RNAseq analysis (Study design #2). The biphasic patterns of expression in both experiments agreed well, suggesting that the ability of these two methodological approaches to identify biphasic DEGs was robust.

**Fig. 7:**
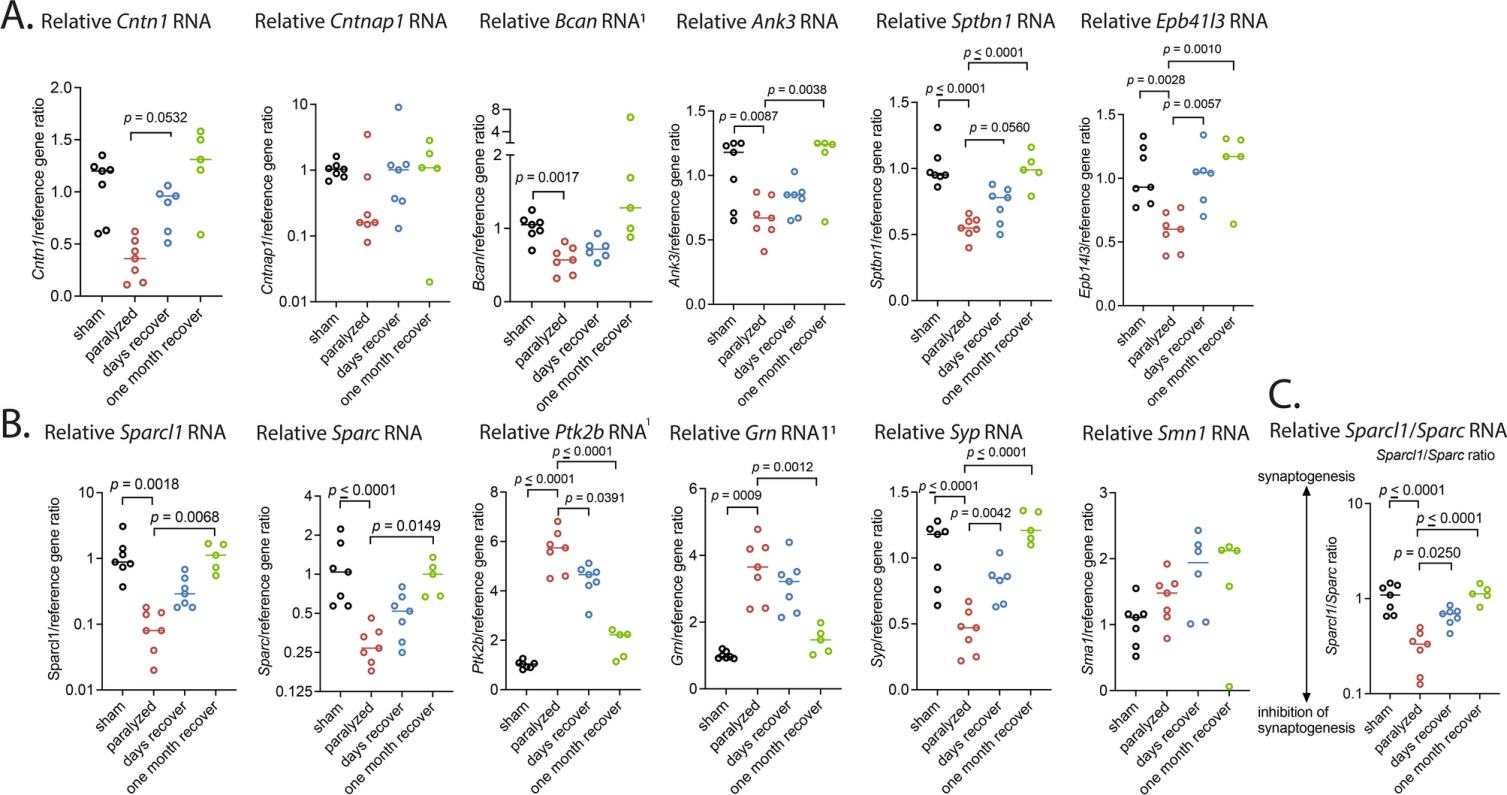
Validation of biphasic DEG expression of genes (Fig. 6) known to affect the structures and functions of the (**A**) nodes of Ranvier and (**B**) synaptic retraction coincident with paralysis followed by recovery. (**C**) synaptogenesis vs. inhibition (*Sparcl1*/*Sparc* ratio)*.* See Fig. 6 for abbreviations. Statistics: One-way analysis of variance with Dunnett’s multiple comparisons test compared to paralyzed animal data. Passes normality test using Kolmogorov test. Due to unequal variances, Brown-Forsythe and Welch ANOVA tests with Dunnett’s T3 multiple comparison tests were performed.

Among the candidate biphasic genes that might affect proximal neuropathy, *Ank3*, *Sptbn1*, and *Epb41l3* had validated biphasic patterns to their expression (Fig. 7A). *Cntn1* and *Bcan* tended to be downregulated during paralysis, although the difference did not reach statistical significance. The RT-qPCR assays confirmed that both *SparcL1* and *Sparc* RNAs are suppressed during paralysis and normalized during recovery (Fig. 7B), but when the ratios of *SparcL1/Sparc* RNA were calculated, the ratios were statistically reduced (*p* ≤ 0.0001) during paralysis reflecting inhibition of synaptogenesis, and then normalization during recovery (Fig. 7C). If protein levels are reflective of these RNA levels, reduced ratio of protein SparcL1/Sparc may lead to inhibition of synaptogenesis, synaptic retraction, and paralysis. Normalization of the Sparcl1/Sparc ratio may then help to restore synaptogenesis and motor function.

*Syp* downregulated-biphasic expression was also validated with RT-qPCR (Fig. 7B). Conversely, *Grn and Ptk2b* RNAs were all up regulated during paralysis, followed by normalization during recovery. *Smn1* appeared to increase as determined by RT-qPCR, but its expression was not biphasic.

## Discussion

We have previously determined that ChAT ir cells, a marker for motor neurons in the spinal cord, were not diminished during paralysis^12^, which suggested that motor neurons do not die to cause paralysis in this ZIKV-induced paralysis model. This prior study also suggested that the motor neurons were not infected because ZIKV immunoreactivity was not colocalized ChAT immunoreactive cells. In this report, we performed more sensitive assays for detection of ZIKV. Virus-specific morphology of particles could not be seen by EM in a small sample of motor neurons of paralyzed mice. However, when fluorescence in situ viral RNA hybridization was employed, 43% of the ChAT RNA-positive neurons were co-localized with *Zikv* RNA expression. Of the three paralyzed mice analyzed, the motor neurons of two mice had colocalized viral RNA, but in the third mouse none of the ChAT RNA-positive neurons counted were colocalized with *Zikv*-RNA. Even though *Zikv* RNA was not detected in recovering mice, immunoreactivity of ZIKV proteins was colocalized in ChAT ir neurons from the spinal cords of some mice that had recovered within days of paralysis, and 1 and 2 months after paralysis. Collectively, these data indicated that some paralyzed mice had infected motor neurons, but the infection of motor neurons were not consistent among all paralyzed mice; hence infection of motor neurons was not strongly correlated with paralysis. Consequently, paralysis may be caused by by-stander effects, such as immune mediated damage of non-motor neurons in infected spinal cords.

To better understand the cellular mechanisms leading to paralysis, we performed bioinformatics analyses on spinal cord RNA seq data to identify DEGs of paralyzed mice compared to sham-infected mice and mice recovering from paralysis. Bioinformatic data generated 12,282 total DEGs between sham-to-paralyzed mice, and paralyzed-to-recovering mice. As with most bioinformatic experiments, we needed an approach to narrow down candidate genes. We reasoned that relevant genes would correlate with changes in disease phenotypes: changes during paralysis and then normalizing during recovery. This approach reduced the number of candidates to 2,058. A similar biphasic DEG approach has been used in a mouse model to identity host responses associated with evolution of influenza A virus quasispecies^38^.

Those DEGs that were altered during paralysis, but normalized during recovery were referred to as “biphasic” DEGs. We expected that biphasic DEGs altered during paralysis, and then normalized during recovery would more likely code for affecter proteins of paralysis or recovery. This filtering processed reduced the number of candidate-DEGs to 7% of total DEGs. Considering that peripheral neuropathy and synaptic retraction may be implicated in ZIKV-induced paralysis^12^, we used multiple gene ontology terms focused on nerve and synaptic functions to identify 8 candidate biphasic DEGs (*Sparcl1*, *Sparc*, *Ptk2b*, *Grn*, *Syp*, *Ank3*, *Sptbn1*, *Epb41l3).*

### Synaptic function

SPARCL1 matricellular protein (SPARC-like 1, SC1, or Hevin) produced by neurosupportive astrocytes directly promotes excitatory synaptogenesis and is downregulated in reactive astrocytes^39^. SPARC produced by astrocytes antagonizes the synaptic activity of SPARCL1. The RNA of both genes are expressed in the top one percentile of biphasic DEGs (data not shown), which agrees with *SparcL1* being one of the highest expressed genes in astrocytes^40^. Consistent with their functional roles for promotion and inhibition of excitatory synapses, paralysis is inversely correlated with *SparcL1:Sparc* transcript ratios and SPARCL1:SPARC protein ratios in the lumbar spinal cord. X-ray crystallography biochemical studies suggest that SPARCL1 proteins promotes synaptic formation by linking presynaptic neurexin-1alpha protein with postsynaptic neuroligin^36^. SPARC protein competes for the binding sites of SPARCL1 to inhibit the formation of excitatory synapses and perhaps to facilitate synaptic retraction during experimental EAE^41^ or ZIKV-induced paralysis. Additionally, SPARCL1 appears to also act independently of neurexins and neuroligins to promote synaptogenesis^42^.

The involvement of SPARL1 and SPARC was previously investigated in EAE mouse models^41^. The investigators used non-remitting and spontaneously remitting autoimmune EAE models, meaning that the animals become paralyzed from immunization of myelin oligodendrocyte glycoprotein peptide and injection of pertussis toxin, and then spontaneously recover^41^. This recovery is the same as with the ZIKV-induced paralysis model of this report. Remarkably, *SparcL1:Sparc* transcript ratios and SPARCL1:SPARC protein ratios inversely correlated with the severity of EAE paralysis, the same as with the ZIKV-induced paralysis model.

Survival motor neuron 1 (*Smn1*) RNA was confirmed by RT-PCR to be increased during paralysis, but *Smn1* RNA was not statistically normalized during recovery. SMN1 protein is involved in mRNA trafficking, spliceosome assembly, synthesis of RNA-binding, and other homeostasis processes^43^. Mutations of the *Smn1* gene lead to alternative splicing and decreased protein, the consequences of which are degenerating alpha motor neurons, progressive muscle weakness, and eventual paralysis^44^ which is referred to as spinal muscular atrophy (SMA). Low levels of full-length *Smn1* RNA are correlates with the severity of the disease^45^. When *Smn1* alternative splicing is reduced with an FDA-approved antisense oligonucleotide treatment that prevents alternative splicing the mortality of infantile-onset disease^46^ and disease phenotypes of late-onset SMA^47^ are significantly improve. The ZIKV-induced paralysis does not involve mutations or alternative splicing of *Smn1,* but the *Smn1* RNA is increased in response to paralysis. The transcriptional increase of *Smn1* RNA^48^ and subsequent protein might be in response to dysfunctional changes to specific cellular processes controlled by Smn1.

Granulin (*Grn*) is another RNA that is confirmed to be increased during ZIKV-induced paralysis and normalized during recovery. The gene codes for progranulin (Pgrn). When translationally produced, Pgrn is cleaved into granulin peptides. Some mutations of *Grn* cause frontotemporal dementia, so human and animal model research has focused on dementia. In these studies, Pgrn functions as a neurotrophic factor and promotes neurite outgrowth of non-motor neuron cells^49–51^, but these data do not preclude Pgrn from similarly promoting motor neuron functions. Missense mutations have also been associated with amyotrophic lateral sclerosis (ALS)^52,53^. Considering this ALS association, one study found that Pgrn and one of the proteolytic fragments of Pgrn (Grn e) promoted motor neuron survival and neurite outgrowth in cell culture^54^. Also, adeno-associated virus-*Grn* partially rescued motor deficits and pathology in *Grn*^+*/−*^ transgenic mice^55^. Another study in a mouse facial nerve-crush model demonstrated that *Grn* gene is induced by nerve injury to restore deficits of motor function of whiskers. Hence, increases of *Pgrn* RNA and Pgrn and Granulin proteolytic fragments during paralysis may be cellular responses to promote motor neuron survival and synaptic formation to assist in recovery from paralysis.

Protein tyrosine kinase 2 beta (*Ptk2b*), highly expressed in the central nervous system, is involved in diverse functions of calcium-induced regulation of ion channels and MAP kinase^56^. It is involved in axon guidance during development^57,58^, synaptic plasticity important for learning and spatial memory^59^, and for astrocyte mobility in response to brain lesions^60^. Ptk2 is associated with some psychological and cognitive pathologies like Alzheimer’s disease^61^, but precedence for any role of Ptk2 in spinal cord functions is not in the published literature.

Synaptophysin (*Syn)* RNA, validated by RT-PCR, was statistically reduced during paralysis and normalized during recovery in spinal cords. The Syn protein is a presynaptic vesicle glycoprotein found in all neurons of the brain and spinal cord that are involved with synaptic transmission. As such, immunostaining of Syn is widely used to quantify synapses^62^. Its function, however, is unclear because animals with inactivated *Syn* gene develop and function normally^63^. Even if Syn protein were to be reduced in the ZIKV-induced paralyzed mice, it will likely have no effect on the function of motor synapses during paralysis or recovery.

### Node of Ranvier and axon initial segment (AIS) functions

Nodes of Ranvier are 1 µm-long gaps between adjacent myelin sheaths, characterized by an accumulation of voltage-gated sodium and potassium channels^64^. These channels are required for rapid and efficient propagation of action potentials along myelinated axons. At paranodes flanking both sides of the nodes, myelinating glial cells interact with axons to form junctions that are essential for node formation and maintenance. Recent studies suggest a common pathogenic mechanism of dysfunction/disruption at the node of Ranvier resulting in a pathophysiologic continuum from rapidly reversible nerve conduction failure to axonal degeneration in axonal form of GBS associated with anti-ganglioside antibodies^65^. Our study validated the biphasic patterns of the expression of genes related to the structures at the nodes and paranodes: *Ank3*, *Sptbn1*, and *Epb41l3* (Fig. 7A). These genes were significantly downregulated in the paralyzed mice after ZIKV infection compared to the sham control and the mice with one-month recovery from paralysis. *Ank3* encodes a scaffolding protein AnkyrinG which is enriched at the nodal axons^26^. AnkyrinG is a critical organizer of the nodes of Ranvier: it binds to voltage-gated sodium and potassium channels, cell adhesion molecule neurofascin 186, and submembranous cytoskeletal protein complex formed by βIV spectrin and αII spectrin^64^. AnkyrinG is also expressed by oligodendrocytes and is enriched on the glial side of paranodes in central nervous system^28^. *Sptbn1* encodes a submembranous cytoskeletal protein βII spectrin which is enriched at paranodes in both axons^33^ and myelinating glial cells^34^. Axonal βII spectrin contributes to the localization of voltage-gated potassium (Kv1) channels at the region next to the paranodes called juxtaparanodes^66^. Glial βII spectrin contributes to the formation and maintenance of paranodal axoglial junctions^34^. *Sptbn1* variants have been reported as the genetic basis of a neurodevelopmental syndrome^67^. *Epb41l3* encodes the cytoskeletal adaptor protein 4.1B which binds the cytoplasmic region of Caspr and form complex with βII and αII spectrin at paranodal axons^64^. Loss of protein 4.1B leads to altered domain organization with reduced clustering of Kv1 channels at juxtaparanodes^68–70^. Thus, the assembly and maintenance of the structures at and near nodes of Ranvier are ensured by multiple mechanisms including AnkyrinG, βII spectrin, and protein 4.1B.

In addition to the nodes of Ranvier, AnkyrinG is accumulated at the AIS, the 20–60 μm long domain located between neuronal soma and axon^71^. Similar to the nodes of Ranvier, voltage-gated sodium channels are highly enriched at the AIS and are responsible for the action potential initiation. The structural characteristics of the AIS—its length and location—are tightly linked to neuronal excitability and firing^72,73^. Alterations in AIS structure or loss of AIS play key roles in the pathophysiology of a wide variety of nervous system diseases such as stroke, epilepsy, and traumatic brain injury^74,75^, although the AIS has not been studied in GBS. AnkyrinG is the master organizer of the AIS assembly and maintenance. Silencing the expression of AnkyrinG dismantles the AIS in cultured hippocampal neurons^29^. The knockout of βII spectrin caused AIS to be significantly disrupted in the hippocampus and cerebral cortex^76^. Loss of protein 4.1B altered distribution of Caspr and Kv1 channels in the domains next to the end of AIS in spinal motor neurons^77^. Since AIS and nodes of Ranvier are essential for action potential initiation and propagation, it is conceivable that the simultaneous reduction of *Ank3*, *Sptbn1*, and *Epb41l3* due to ZIKV infection can alter functions and structures of these excitable axonal domains.

The observation that two of seven paralyzed mice did not have infectious virus in their homogenized spinal cords (Fig. 5A) suggests that this may represent parainfectious paralysis, i.e., extra-spinal cord viral infection occurring before the onset of paralysis and that ensuing infectious myelitis of the spinal cord may not contribute to the paralysis. Therefore, future studies should determine if this model is relevant by investigating various biomarkers and pathology of human immune-mediated myelitis or GBS.

Previous electrophysiological studies, including F-wave analysis, in this mouse model^12^ suggests the presence of neuropathy in nerve roots. This is relevant since neuropathy in roots is associated with early onset of GBS^78,79^. The pathophysiology of the nodes of Ranvier should be investigated because we identified biphasic DEGs that are known to affect the structure and function of nodes of Ranvier. Given the importance of the nodes of Ranvier in regulating action potential propagation, it is not surprising that disruption of nodes and associated structures plays key roles in various neurological diseases^80,81^. In the axonal form of GBS associated with anti-ganglioside antibodies, human and experimental data suggest a common pathogenic mechanism of dysfunction/disruption at the node of Ranvier resulting in a pathophysiologic continuum from rapidly reversible nerve conduction failure to axonal degeneration^65^. In human axonal GBS, an early pathological feature includes the elongation of the nodes of Ranvier with deposition of complement products in the ventral roots^15,16^. Therefore, the length of nodes of Ranvier and complement deposition of nerve roots should be determined. Other GBS pathophysiological features could also be measured: elevated CSF protein without corresponding increased cell counts (albuminocytologic dissociation), increased inflammatory immune cells beginning at the nerve roots and descending along peripheral nerve fibers, and presence of anti-ganglioside antibodies.

The primary contribution of this study is the identification of candidate genes that may be involved in the causation or recovery of ZIKV-induced paralysis. The biphasic expression levels of these genes will need to be validated with measurement of the respective proteins. Subsequently, pharmacological or gene altering experiments will need to be done to ultimately identify the mechanism of the reversible paralysis.

## Methods

### Ethics declaration

This study was conducted with the approval of the Institutional Animal Care and Use Committee of Utah State University and in accordance with the AAALAC-accreditation (reference file #000649), with the National Institutes of Health Guide for the Care and Use of Laboratory Animals (Assurance no. A3801-0), and with reporting criteria of ARRIVE guidelines. The endpoint for humane euthanasia to minimize pain and distress was 30% or greater weight loss. CO_2_ euthanasia was used according to the Guidelines for the Euthanasia of Animals published by the American Veterinary Medicine Association (AVMA).

### Animals and study designs

#### Study design #1

At 4.7 months of age, 16 male and 16 female IFNAR^−/−^ mice (originally purchased from The Jackson Laboratory, MMRRC Stock No: 32045-JAX) were injected subcutaneously (s.c.) in the inguinal area on both sides with 600 pfu/mouse of ZIKV (ZIKA PRVABC59, P1, 2/12/16-P2) in volumes of 0.1 mL (Table S4). Two IFNAR^−/−^ mice were injected with sham (cell culture homogenate). The VPS was obtained daily beginning day 8 after viral challenge. Lumbosacral cords from 2 sham-infected, 3 ZIKV-infected mice before paralysis (pre-paralysis), 4 paralyzed mice (VPS = 5 or 6) on both hind limbs, and 5 recovered mice with VPS scores on either side were prepared for fluorescence in situ hybridization by RNAScope™ analysis.

#### Study design #2

At a mean age of 5.3 months, 88 male and 20 female IFNAR^−/−^ mice were injected subcutaneously (s.c.) in the inguinal area on both sides with 600 pfu/mouse of ZIKV (ZIKA PRVABC59, P1, 2/12/16-P2) in volumes of 0.1 mL (Table S5). Six IFNAR^−/−^ mice were injected with sham (cell culture homogenate). The VPS was obtained daily beginning at day 8 after viral challenge. Lumbosacral cords from sham-infected, paralyzed mice (VPS = 5 or 6) on both hind limbs, mice at 1 to 3 days, at 1 month, and at 2 months after recovery from paralysis were necropsied for fluorescence in situ hybridization by RNAScope™ analysis, immunohistochemistry, or RNAseq analysis.

#### Study design #3

At ages of 4.2 to 5.2 months, 62 male and 68 female IFNAR^−/−^ mice were injected s.c. in the inguinal area on both sides with 1,340 pfu/mouse of ZIKV (ZIKA PRVABC59, P1, 2/12/16—P2) in volumes of 0.1 mL (Table S6). Seven IFNAR^−/−^ mice were injected with sham (cell culture homogenate). The VPS was obtained daily beginning 8 days after viral challenge. Lumbosacral cords from sham-infected (7 each), paralyzed mice (VPS = 5 or 6) (7 each) on both hind limbs, mice at 1 to 3 days (7 each), at 1 month (5 each), and at 2 months (5 each) after recovery from paralysis were necropsied for validation of biphasic DEGs by RT-qPCR, infectious ZIKV titers, and relative gene expression of *Zikv* RNA by RT-qPCR.

### Bioinformatics

#### Spinal cord preparation

Spinal cords were dissected from the vertebrate column anterior between the hind limbs and posterior the medulla oblongata. Cords from mice of Study design #1 were prepared to enrich for motor neurons^82,83^. Cords were cut into small pieces in papain (2 mg/mL of HBSS with glucose and sucrose, Sigma-Aldrich) and digested for 30 min in a tissue culture incubator at 37 °C. The tissue in cold medium was gently homogenized slowly using a cold pestle tissue grinder. The suspension was centrifuged at 100×*g* for 5 min (4 °C). The supernatant was suspended in 5 mL cold L-15 medium (Thermo Fischer Scientific) and overlaid over a LSM density solution (1.06 g/mL density) (Wisent Bioproducts, cat # 305-010-CL). The sample was centrifuged at 425×*g* for 10 min in a swinging bucket centrifuge at 4 °C to enrich for motor neurons. A small portion of cells was smeared on a glass slide and stained with anti-ChAT antibody. Far less than 50% of cells were ChAT-reactive. Trizol™ RNA extraction and ethanol precipitation were performed.

Spinal cords from Study designs #2 and #3 were not prepared to enrich for motor neurons. The RNAs from cords homogenized in MEM without papain digestion were extracted using Trizol™ RNA extraction and ethanol precipitation.

#### Next-generation sequencing

RNA quality was analyzed using the Agilent TapeStation 2200. Ribosomal RNA was depleted using the QIAseq FastSelect -rRNA HMR kit (334385) and sequencing libraries were prepared with the NEBNext Ultra II Directional RNA Library Kit (E7765L) for Illumina following the manufacturer’s protocol. Libraries were sequenced on an Illumina NextSeq 500 using the 75 cycle v2.5 sequencing kit (2024904) and base-calling was performed using bcl2fastq v2.20.

#### Raw data processing and gene quantification

pySeqRNA (https://bioinfo.usu.edu/pyseqrna/)^84^ was used to process the raw data sequences. pySeqRNA is a Python tool for analyzing large-scale transcriptome data. We used the pySeqRNA with default settings for transcriptome analysis. The *quality_check* module was used to assess the quality of raw sequencing reads. The *quality_check* module used FASTQC (https://www.bioinformatics.babraham.ac.uk/projects/fastqc/) in the backed to check read quality. The adapter, poly-N, and low-quality reads were removed using the default trimming tool in Trim Galore (https://www.bioinformatics.babraham.ac.uk/projects/trim_galore/) in *quality_trimming* module, followed by an after-trim quality check using *quality_check*. Cleaned reads were mapped on the Mus musculus genome retrieved from (https://uswest.ensembl.org/Mus musculus/Info/Index) using the STAR aligner from aligner module^85^. The featureCounts^86^ from *quantification* module was used to generate raw read counts. Using the *normalize_counts* module, the raw read counts were standardized to Reads Per Kilobase Million (RPKM).

#### Identification of differentially expressed genes (DEGs)

The *differential expression* module of pySeqRNA was used to calculate the differential expression of genes. DESeq2^87^ wrapper was used to perform differential expression analysis of two conditions/groups of samples. DESeq2 provides statistical routines for identifying differential expression in digital gene expression data based on the negative binomial distribution model. To account for the false discovery rate, the P-values were adjusted using the Benjamini and Hochberg method. pySeqRNA internal *degFilter* function was used to filter differentially expressed genes with FOLD change 2 and FDR ≤ 0.05.

#### Functional enrichment analysis of DEGs

pySeqRNA was used to perform functional enrichment of differentially expressed genes. The *pathway* module was used to find enriched KEGG pathways in DEGs, and the *gene_ontology* module was used to find enriched gene ontology terms in DEGs. In the backend, pySeqRNA uses BioMart for Gene Ontology (GO) enrichment and http://bioinfo.usu.edu/pyseqrna-api/ for KEGG (Kyoto Encyclopedia of Genes and Genomes) pathway enrichment. Using gene ontology (GO) term analysis, transcripts were classified into three major functional groups: molecular functions, biological processes, and cellular components.

#### Extraction of common reads

Further, the common reads between uninfected samples and recovered samples were identified using an in-house Python script developed in the Kaundal Lab. The parameters considered for common reads includes sequence match, sequence length, ignoring case of letters in headers, etc. These common reads were further processed with pySeqRNA for analysis.

### In situ RNA hybridization using RNAscope™

#### Animals and tissue preparation

Details of the mouse strain, sex, and age are described in Study Designs #1, and #2 (“Methods” section). Mice were fixed by cardiac perfusion with freshly prepared 4% paraformaldehyde (PFA) and the lumbosacral spinal cords (L3-L5) were dissected and fixed overnight at 4 °C. The tissues were immersed in 10% sucrose followed by 20% and 30%, and then frozen in optimal cutting temperature embedding media with liquid nitrogen (ACD Technical Note: Preparing Fixed Frozen Tissue for the RNAScope™ for 18 h Fluorescent Multiplex Assay). They were stored in an airtight container at − 80 °C until cryosectioning. Spinal cord specimens were sectioned coronally on a cryostat at 12 µm at − 20 °C and thaw-mounted to Fisher SuperFrost-Plus microscope slides. Slides were frozen at − 80 °C until use in in situ hybridization assays.

#### Chromagenic in situ hybridization (ISH)

RNAScope™ (ACD Bio, Inc) was used to visualize the RNA for *Zikv* and *Chat* in the ventral spinal cord in all specimens according to manufacturer’s protocols for fixed frozen tissue. The list of reagents is shown (Table S1). The slides were in a humidified oven (HybEz, ACD) to 40 °C for 30 min. The slides were dehydrated with serial 5 min washes in 50%, 70%, and 100% ethanol before being treated for 10 min with hydrogen peroxide. Next, slides were submerged in boiling RNAScope™ for 30 min 1X Target Retrieval solution, followed by two rinses in distilled water and a 3-min rinse 100% ethanol. After drying, individual tissue slices on each slide were surrounded by a hydrophobic barrier using an RNAScope™ ImmEdge pen. Each slice was treated with RNAScope™ Protease III for 30 min at 40 °C to further unmask antigens. The slides were rinsed twice in fresh distilled water prior to incubation with target probes. Each specimen was processed in triplicate sets of adjacent sections: one section was incubated in experimental probes (a combination of ChAT and Zika probes), one section was incubated in the negative control probe (*dapb*, bacterial genes), and one section was incubated in the positive control probes (*PPIB* and *POL2RA*, two mammalian housekeeping genes). Probes were mixed in a hybridization solution at a ratio of 1:50 of C2 probe to C1 probe. The probes were allowed to hybridize for two hours at 40 °C in a humidified chamber. After hybridization, the slides were rinsed twice in 1X wash buffer and stored in 5X SSC overnight. The next day, the slides were rinsed in wash buffer and then processed through the ten RNAScope™ amplification steps, one at a time, as well as the red and green chromogenic probe labeling steps. Cell nuclei were then counterstained by submerging the slides in 50% Gill’s Hematoxylin-I for 30 s. Finally, the slides were rinsed in fresh distilled water, dried at 60 °C for 15 min, cooled, and cover-slipped using VectaMount (Vector Laboratories, Burlingame, CA).

Analytical quantitation of chromogenic ISH was performed^88^. Keyence BZ-X800 microscope (Keyence Corporation of America) capable of brightfield imaging was used to capture 20X images from all specimens. Our quantification approach used the hue of each of the chromogenic stains (red/magenta and green/cyan). A mask of each chromogenic stain was created from one representative image containing both red and green signal was manually created using the Hybrid Cell Count function in the Keyence Corporation’s BZ-X800 Analyzer software (version 1.1.2.4). Each hue-based mask is made by incrementally adding the hues of interest by selecting individual pixels on one representative image and toggling the sensitivity and tolerance of the mask settings until the pattern of quantified pixel area aligns with true pattern of that colored stain on the image being quantified. We then saved those settings as a macro and applied it to all images to standardize the quantification approach. All the resulting masks generated by the saved macro were visually checked to ensure that the quantified pixel area aligned with the visual distribution of signal stain. In a few cases where the expression levels were absent or low, causing issues with the resulting masks, a new mask was generated for that problematic image individually, or zeros were included in cases where there was truly no signal to quantify (for example, for green Zika mRNA in uninfected sham specimens). The total pixel area covered by each probe’s hue was included in our final dataset. The parametric data were statistically analyzed using GraphPad Prism (version 10.1.1). The specific statistical tests used are stated in the figure captions.

#### Fluorescent in situ hybridization

RNAScope™ Multiplex Fluorescent v2 Assay (Table S2) was used according to the manufacturer’s instructions (Advanced Cell Diagnostics, Inc) for fixed, frozen brain tissue. Slides were heated in a humidified 4 °C oven for 30 min and then dehydrated in ascending concentrations of ethanol. Endogenous peroxidases were quenched with H_2_O_2_ for 10 min at ambient temperature. Slides were boiled for 10 min in the Target Retrieval Reagent and then incubated for 30 min at ambient temperature in RNAscope Protease III before probe hybridization. We used a pooled duplex probe containing a probe targeting *Zika* mRNA and a probe targeting mouse *ChAT* mRNA. Probes were hybridized for 2 h at 40 °C in a humidified 40 °C oven. After hybridization, slides were washed in wash buffer (two, 2-min washes) and stored in a 5X saline sodium citrate stop solution overnight at ambient temperature. Probes were amplified according to manufacturer’s instructions then visualized with distinct Opal dyes: Opal 520 (green; equivalent to Cy3/GFP) for *ChAT* and Opal 570 (red; equivalent to Cy5) for *Zika* (Table S2). Tissues were then counterstained with DAPI for 30 s and immediately cover-slipped with Prolong Gold. Confocal microscopy is described^12^.

### RT-qPCR and infectious viral assays

Lumbosacral cords (Study design #3) were necropsied for qRT-PCR of *Eef1a1* plus *Gapdh* as reference genes. The primers, and probes, reaction conditions were developed with Integrated DNA Technologies (IDT) online programs. We used 2× PrimeTime one-step RT-qPCR Master Mix (IDT) and QuantStudio 5™ Real-Time PCR instrument and software. The primers and probes are listed and the method for selection is described (Table S3).

#### Infectious cell culture assay

The viral titers in homogenized lumbosacral cord were assayed using the virus yield assay^89^ where a specific volume was added to the first tube of a series of dilution tubes. Serial dilutions were made and added to Vero cells. Five days later, the viral cytopathic effect (CPE) was used to identify the endpoint of infection. Four replicates were used to calculate the 50% cell culture infectious dose (CCID_50_) per mL of serum or gram of tissue.

To amplify undetected virus, the spinal cord homogenates (300 µL) of viral titers below the limits of detection were added to 6-well tissue culture plates seeded with Vero 76 indicator cells (3 mL total volume). At 6 days, 300 µL of cell culture supernatant were passaged to a second set of 6-well tissue culture plates. At 6 days, supernatants were quantified by the assay above.

#### Footprint acquisition

An enclosed T-shaped runway with an open end at the long end was built from white cardboard (3.75 in. high × 3.65 in. wide × 36 in. length of runway). Mice were placed in the straight end of the runway. They were trained to walk to the dark compartment by prodding them with a probe. After training, a white paper was placed in the bottom of the straight runway. While holding the mouse by the scruff of the neck, the ventral and dorsal hind paws were thoroughly painted with blue ink up to the hock and on all 5 toes. The front paws were also painted. The mouse was released at the opening of the runway. If necessary, the mouse was prodded to walk/run down the runway. Images of paralyzed mice for Study design #1 (Fig. S2) and Study design #2 (Fig. S3) are shown.

## Supplementary Information

Supplementary Information.

## Data Availability

All the sequencing reads generated from Illumina HiSeq RNA-Seq are available in the NCBI SRA: SRR30148281 to SRR30148293 (https://www.ncbi.nlm.nih.gov/Traces/study/?acc=SRP524499&o=acc_s%3Aa). The BioProject accession number is PRJNA1144966. All other datasets supporting this study are included in the article and its supplementary material. Data are available from the corresponding author on reasonable request. All resources are available upon request from the corresponding author or from the stated commercial suppliers.
