# Supplementary material for: Identification of candidate genes involved in Zika virus-induced reversible paralysis of mice
Source: Sci Rep. 2025 Jan 23;15:2926. doi: 10.1038/s41598-025-86475-0 (PMC11757732; doi:10.1038/s41598-025-86475-0)
Supplement: Supplementary file 1 — Supplementary Information. [file 41598_2025_86475_MOESM1_ESM.docx]

**Supplemental information**


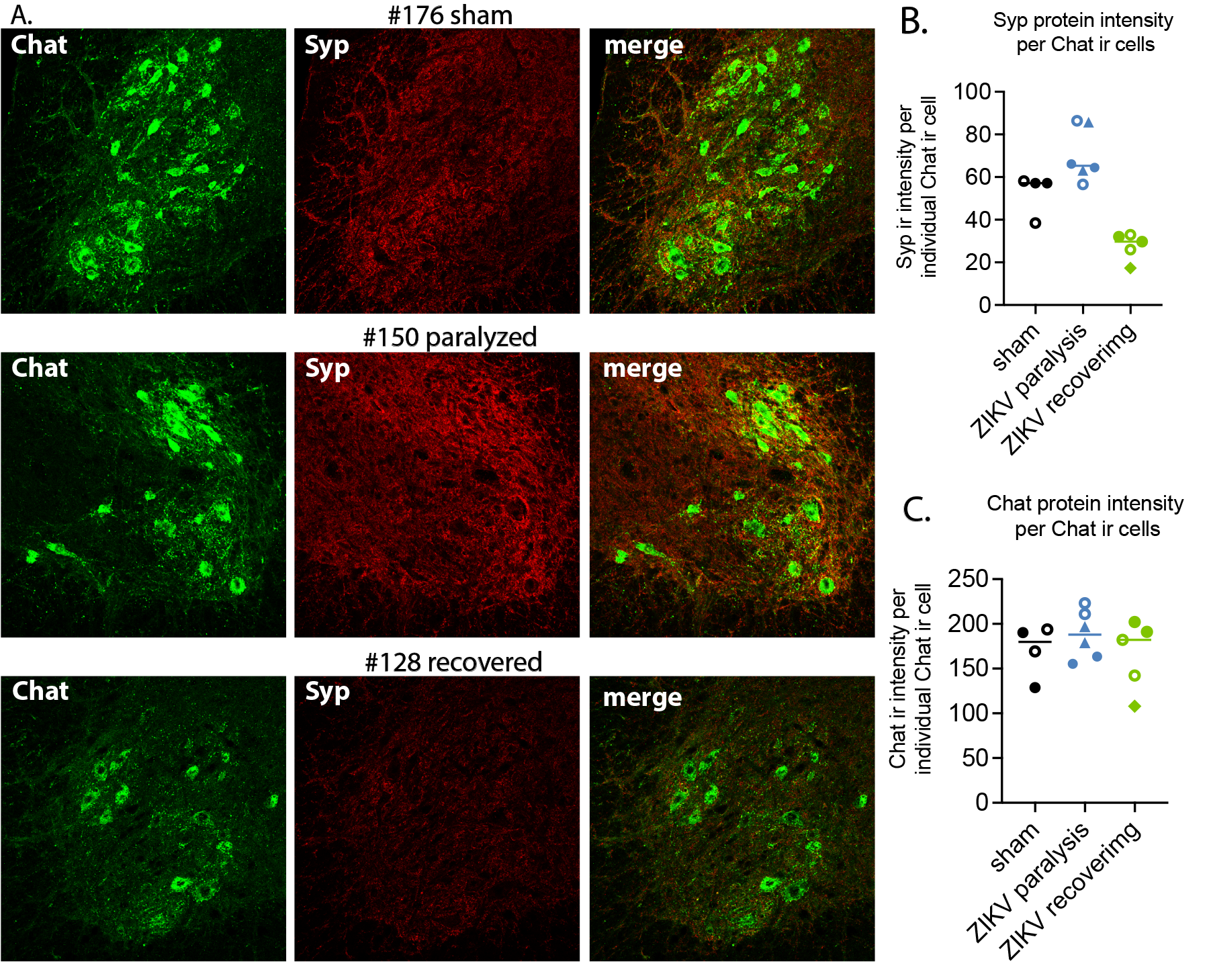


**Figure S1.** Modulation of pre-synaptic synaptophysin (Syp) protein immunoreactivity in outer membrane area of ChAT ir cells in mice with motor deficits and in recovering mice compared to sham-infected mice. Male IFNAR mice (4.0 to 4.2 weeks old) were challenged s.c. in the inguinal area on one side with 2,000 pfu/mouse of ZIKV (ZIKA PRVABC59, P1, 2/12/16 - P2) in 0.1 mL volume. The viral paresis score (VPS) was obtained daily beginning 2 days after viral challenge. Lumbosacral cords from sham-infected mice (VPS = 0), ZIKV-induced motor deficit mice mice (VPS = 2.5 to 6), and from mice after disease signs at day 15 were fixed. Histological methods are described ^10^. Histological sections (12 µm thick) were analyzed by confocal microscopy for intensity of Chat (green) and synaptophysin (red). The same symbols are sections from the same mouse. **A)** Examples of a sham-infected mouse (#176), ZIKV-induced paralyzed mouse on one leg (#150), and a mouse at day 15 after recovery (#128) illustrate increase of Syp in mice with motor deficits followed by a decrease in recovering mice. **B)** Mean ChAT intensity per motor neuron. **C)** Mean synaptophysin intensity in ChAT immunoreactive cells. Each data point represents the mean of lumbosacral spinal cords on either the right or left per mouse. Eight to 22 fields of each data point at 20x were measured by ImageJ (image J method below).

**Figure S2.** Footprint assay images of paralyzed mice of Study design #2.

**
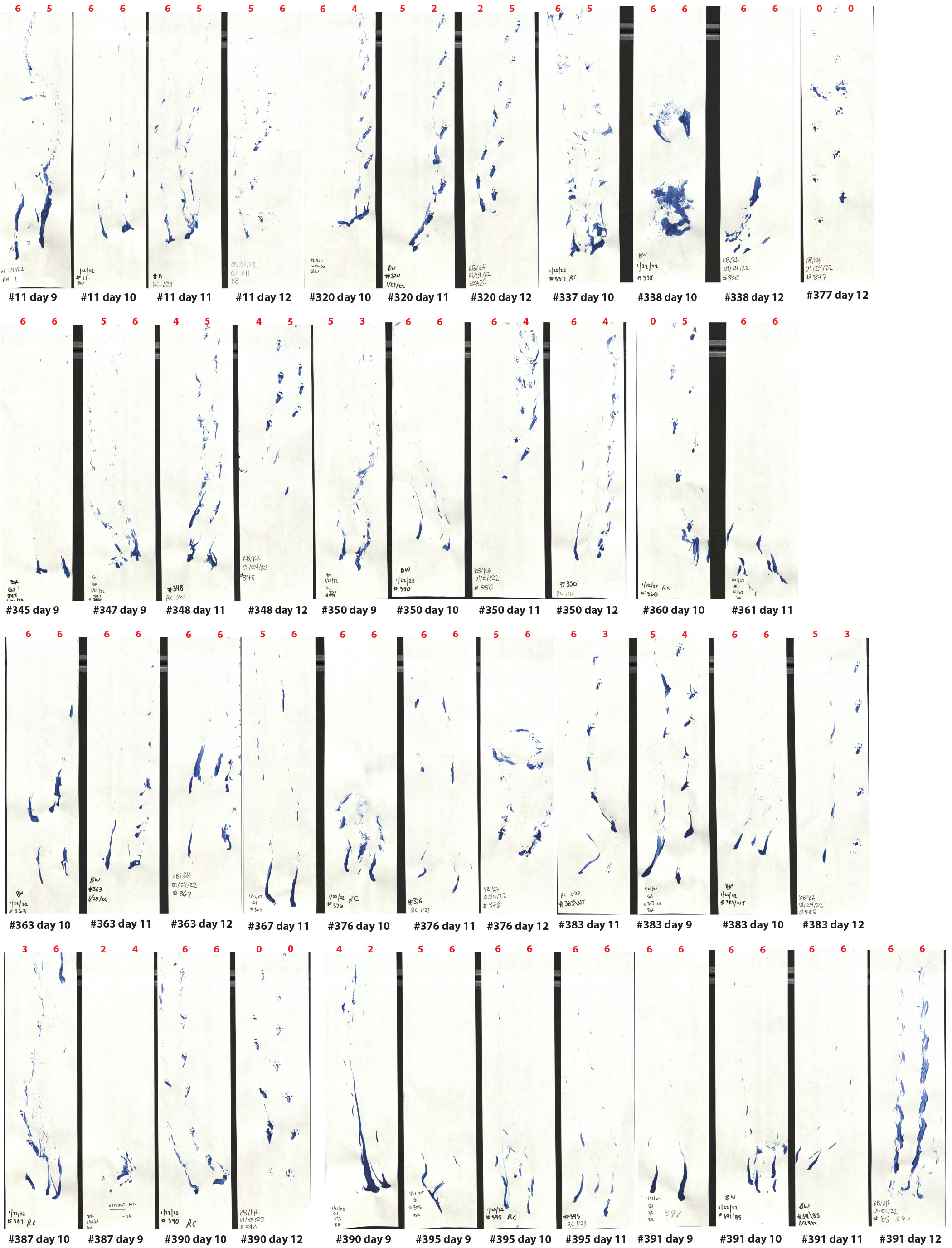
**

**Figure S3.** Footprint assay images of paralyzed (left column) and samples of recovered (right column) mice of Study design #3.


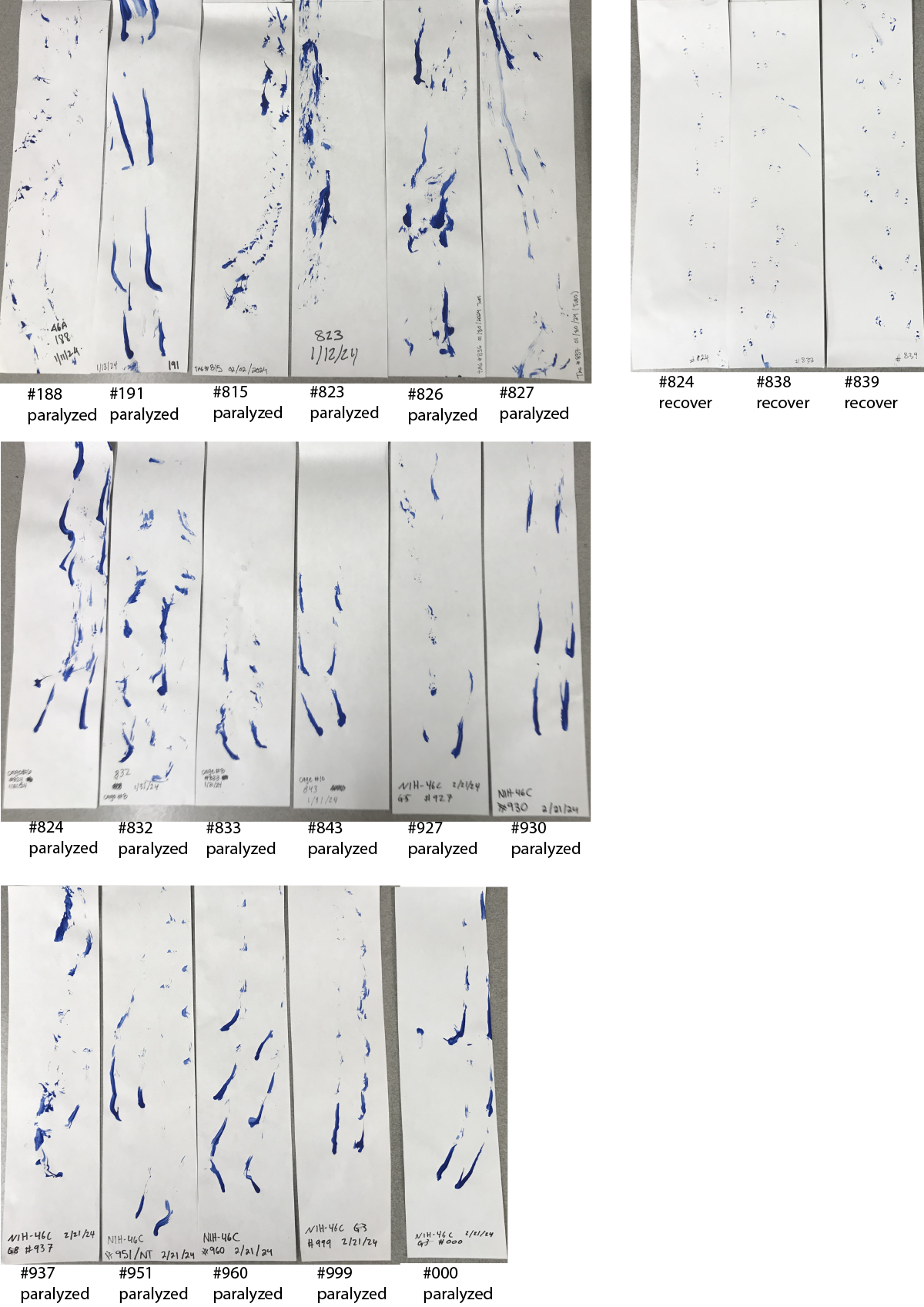


| **Table S1**. List of RNAScope™ reagents for chromogenic *in situ* hybridization (chromogenic ISH). | | |
| --- | --- | --- |
| Catalog # | Description | |
| 408731 | RNAscope™ Probe - Mm-Chat - Mus musculus ChAT mRNA (channel C1) | |
| 408731-C2 | RNAscope™ Probe - Mm-Chat-C2 - Mus musculus ChAT mRNA (channel C2) | |
| 1070921-C2 | RNAscope™ Probe - V-ZIKV-pp-C2Zika virus PRVABC59 complete genome (channel C2) | |
| 1070921-C1 | RNAscope™ Probe - V-ZIKV-pp-C2Zika virus PRVABC59 complete genome (channel C1) | |
| 322430 | RNAscope™ 2.5 HD Duplex Reagent Kit using HRP (Green) and Fast Red chromogenic stains. Each kit contains: RNAscope Duplex Detection Kit (PN 322500) RNAscope Pretreatment Reagents (PN 322000, 322330), RNAscope Wash Buffer (PN 310091). | |
| 321651 | RNAscope™ 2.5 Duplex Positive Control probe with PPIB (C1 channel) and Polr2a (in C2 channel). | |
| 320751 | RNAscope™ 2-plex Negative Control Probe DapB | |
| 321584 | Vector Labs Vectamount | |
| 310018 | ImmEdge Hydrophobic Barrier Pen | |
| 322337 | RNAscope™ Protease III | |
| AB144P | Sigma-Aldrich, goat anti-choline acetyltransferase antibody, used at 1/100 in 0.1 Triton X-100 + 1% BSA |  |
| 0308-001 | IBT Bioservices, Rabbit anti-ZIKA polyclonal antibody to envelope glycoprotein peptide, 1:500 in 0.1 Triton X-100 + 1% BSA |  |
| A-11057 | ThermoFisher, donkey anti-goat IgG Alexa Fluor™ 568 secondary antibody at 1:500 |  |
| A-21206 | ThermoFisher, donkey anti-rabbit IgG Alexa Fluor™ 488 secondary antibody at 1/250 |  |

| **Table S2.** List of RNAScope™ reagents for fluorescence *in situ* hybridization (fluorescence ISH). | | | |
| --- | --- | --- | --- |
| Catalog # | | Description | |
| 408731 | RNAscope™ Probe - Mm-Chat - Mus musculus ChAT mRNA (channel C1) | |  |
| 1070921-C2 | RNAscope™ Probe - V-ZIKV-pp-C2Zika virus PRVABC59 complete genome (channel C2) | |  |
| 426521-C3 | RNAscope™ Probe – Mm-/Syp-C3 – Mus musculus synaptophysin (Syp) mRNA (channel C3) | |  |
| 323136 | RNAscope™ Intro Pack for Multiplex Fluorescent Reagent Kit v2- Mm recommended for new users getting started with RNAscope assay on mouse samples. It includes required materials such as: RNAscope™ Multiplex Fluorescent Reagents Kit, FFPE Control Slide Pack -Mouse 3T3 Cell Pellet (PN 310023), Hydrophobic Barrier Pen (PN 310018), RNAscope™ 3-plex Positive Control Probe_Mm(PN 320881), RNAscope™ 3-plex Negative Control Probe (PN 320871) and RNAscope™ Probe Diluent (300041). Users can use the control slides and the probes, to get familiar with the assay workflow and staining results. Opal fluorophores from Akoya Biosciences must be purchased separately (Opal 520, PN FP1487001KT; Opal 570, PN FP1488001KT; Opal 690, PN FP1497001KT). | |  |
| 321651 | RNAscope™ 2.5 Duplex Positive Control probe with PPIB (C1 channel) and Polr2a (in C2 channel). | |  |
| 320751 | RNAscope™ 2-plex Negative Control Probe DapB | |  |
| 321584 | Vector Labs Vectamount | |  |
| 322337 | RNAscope™ Protease III | |  |
| AB144P | Sigma-Aldrich, goat anti-choline acetyltransferase antibody, used at 1/100 in 0.1 Triton X-100 + 1% BSA | |  |
| FP1487001KT | Akoya Biosciences, OPAL 520 Reagent pack | |  |
| FP1488001KT | Akoya Biosciences, OPAL 570 Reagent pack | |  |
| FP1497001KT | Akoya Biosciences, OPAL 690 Reagent pack | |  |
| 0308-001 | IBT Bioservices, Rabbit anti-ZIKA polyclonal antibody to envelope glycoprotein peptide, 1:500 in 0.1 Triton X-100 + 1% BSA | |  |
| A-11057 | ThermoFisher, donkey anti-goat IgG Alexa Fluor™ 568 secondary antibody at 1:500 | |  |
| A-21206 | ThermoFisher, donkey anti-rabbit IgG Alexa Fluor™ 488 secondary antibody at 1/250 | |  |

| **Table S3.** Primer/probe sets and methods of selection^a^ | | | |
| --- | --- | --- | --- |
| Gene transcript | NCBI sequence for primer design | Position | Sequence |
| *Eef1a1* | NM 010106.2 | forward | GCTGGAGCCAAGTGCTAATA |
|  |  | probe | /56-TAMN/TGGTTCAAGGGATGGAAAGTCACCC/3IAbRQSp/ |
|  |  | reverse | TGGTGGTAGGATACAATCCAAAG |
| *Gapdh* | NM 001289726.2 | forward | GGAGAAACCTGCCAAGTATGA |
|  |  | probe | TCAAGAAGGTGGTGAAGCAGGCAT |
|  |  | reverse | TCCTCAGTGTAGCCCAAGA |
| *Ank3* | NM 170728.2 | forward | CAATGCCAAAGCCCTGAATG |
|  |  | probe | /56-FAM/AACCGCATC/ZEN/CGAGTAATGGAACTCC/3IABkFQ/ |
|  |  | reverse | GACTCGGTTACGGCTTGAATAG |
| *Epb41l3* | NM_013813.2 | forward | CGGAAACCCACAGAGTTCATA |
|  |  | probe | AGAGCTGGGTGCAGAAACTGGAAA |
|  |  | reverse | GATTCCACCTCTGCTTCTACAG |
| *Bcan* | NM_001109758.2 | forward | GTCAAGACCCTCTTCCTCTTTC |
|  |  | probe | /56-FAM/CCGCTTCAA/ZEN/TGTCTACTGCTTCCGA/3IABkFQ/ |
|  |  | reverse | AATGGCCTCAAGTCCATCTG |
| *Cntn1* | NM 001159647 | forward | CTACTGTGACAGGCTACAAGATAC |
|  |  | probe | /56-FAM/ACGGCAAGC/ZEN/TGTTCTCAACCCATA/3IABkFQ/ |
|  |  | reverse | CTCGAACCTCGACAACATACTC |
| *Cntnap1* | NM_016782.2 | forward | TAACTTCCGTGGCTGCATAG |
|  |  | probe | /56-FAM/CAGAAATGG/ZEN/CAGTGATGCGCCATT/3IABkFQ/ |
|  |  | reverse | GCAACGGAAAGCCACATTAC |
| *Gyn* | NM_008175.5 | forward | GGGCATTTCTGCCATGATAAC |
|  |  | probe | /56-FAM/TAAAGACAG/ZEN/TGCAGGAGTCTGGGC/3IABkFQ/ |
|  |  | reverse | CAACAGTGACGTCCATCTCTAC |
| *Ptk2b* | NM_001162366.2 | forward | GAGAACATGGCTGACCTCATAG |
|  |  | probe | /56-FAM/AGAGCCCTT/ZEN/ATGTTCTCCTTGCAGC/3IABkFQ/ |
|  |  | reverse | GTTCCTCTTCTCACCATCTTTCT |
| *Sma1* | NM 011420.2 | forward | CAGAACAGAACACTCAGGAGAA |
|  |  | probe | /56-FAM/TCGCTCAGA/ZEN/AGTAAAGCACACAGCA/3IABkFQ/ |
|  |  | reverse | GGTGGTGGAGGAAGAAATGA |
| *Sparc* | NM_001290817.1 | forward | CATCAAGGAGCAGGACATCAA |
|  |  | probe | /56-FAM/ATCTAAGTT/ZEN/CACGCCTCCTGCTGC/3IABkFQ/ |
|  |  | reverse | GGACACATCAGAGGGAGAGA |
| *Sparcl1* | NM_001359014.1 | forward | GCAAGGAACACATTCCACATAC |
|  |  | probe | /5SUN/TTTCCCTTC/ZEN/TTGGTCCTGCTGCTC/3IABkFQ/ |
|  |  | reverse | CCTTCTCATCAGTGTCCTTCTG |
| *Sptbn1* | NM 175836.2 | forward | AGCGTGTAAGAGGTGTGAATG |
|  |  | probe | /56-FAM/CCCATCTGT/ZEN/TGCAAACTTCTGGGC/3IABkFQ/ |
|  |  | reverse | GGCAACACGGTCTCGAATTA |
| *Syp* | NM 009305.2 | forward | AGTGGGTCTTTGCCATCTTC |
|  |  | probe | /56-FAM/AGTGTGCCA/ZEN/ACAAGACGGAGAGTG/3IABkFQ/ |
|  |  | reverse | CAGCCTGAATGGGTACTCAAA |
| *Zikv* | NM_016782.2 | forward | GAGTGTGATCCAGCCGTTATT |
|  |  | probe | /56-FAM/AGCTGTTAA/ZEN/GGGAAAGGAGGCTGT/3IABkFQ/ |
|  |  | reverse | CAGCCTCCATGTGTCATTCT |
| ^a^Five primer/probes sets were selected from the NCBI sequence listed using IDT’s (Integrated DNA Technologies, Inc.) PrimerQuest Tool. The top candidate was selected by verifying that the PCR product covered an exon junction site, had the most favorable delta G value with the probe to minimize intermolecular annealing, and that the probe detected all spice variants (BLAST). The probes for the reference genes (*Eefa1a1* and Gadph) were labeled with TAMRA. All other probes were labeled with FAM, except for *Sparcl1* that was labeled with SUN because it was used in a duplex reaction with *Sparc* labeled with FAM. | | | |

| **Table S4.** Inventory of necropsied mice from study #1. Expt # NIAW-947. | | | | | | |
| --- | --- | --- | --- | --- | --- | --- |
| Group | Mouse # | Day of necropsy | Day of last paralysis | VPS at last paralysis | Necropsy day minus last paralysis day | Procedures |
| sham | 333 | 10 | NA^a^ | NA^a^ | NA^a^ | chromogenic ISH |
| sham | 334 | 10 | NA^a^ | NA^a^ | NA^a^ | chromogenic ISH |
| before^b^ | 304 | 9 | NA^a^ | NA^a^ | NA^a^ | chromogenic ISH |
| before^b^ | 307 | 9 | NA^a^ | NA^a^ | NA^a^ | chromogenic ISH |
| before^b^ | 312 | 9 | NA^a^ | NA^a^ | NA^a^ | chromogenic ISH |
| paralyzed | 302 | 9 | 9 | 6, 6 | 0 | chromogenic ISH |
| paralyzed | 303 | 9 | 9 | 6, 6 | 0 | chromogenic ISH |
| paralyzed | 313 | 9 | 9 | 6, 6 | 0 | chromogenic ISH |
| paralyzed | 314 | 9 | 9 | 6, 6 | 0 | chromogenic ISH |
| paralyzed | 315 | 9 | 9 | 6, 6 | 0 | chromogenic ISH |
| days^c^ | 301 | 12 | 9 | 5, 1 | 3 | chromogenic ISH |
| days^c^ | 305 | 12 | 10 | 6, 4 | 2 | chromogenic ISH |
| days^c^ | 308 | 15 | 12 | 3, 6 | 3 | chromogenic ISH |
| days^c^ | 318 | 13 | 11 | 6, 5 | 2 | chromogenic ISH |
| days^c^ | 329 | 15 | 11 | 6, 6 | 4 | chromogenic ISH |
| ^a^ Not applicable  ^b^ Infected mice necropsies before paralysis was expected to occur.  ^c^ Approximate time after paralysis that necropsy was performed.  ^d^ Freshly dead, was not going to recover. Rare animals in this model progress to severe disease and die.  ^e^ Highest VPS was 3.  ^f^ Stopped reading at day 15 until resumption of reading at day 37 when fully recovered (VPS equal to 0 or 1).  ISH – *in situ* RNA hybridization | | | | | | |

| **Table S5.** Inventory of necropsied mice from Study #2. Expt # NIAW-984. | | | | | | |
| --- | --- | --- | --- | --- | --- | --- |
| Group | Mouse # | Day of necropsy | Day of last paralysis | VPS at last paralysis | Necropsy day minus last paralysis day | Procedures |
| sham | 301 | 9 | NA^a^ | NA^a^ | NA^a^ | IHC |
| sham | 302 | 9 | NA^a^ | NA^a^ | NA^a^ | IHC |
| sham | 303 | 13 | NA^a^ | NA^a^ | NA^a^ | chromogenic ISH |
| sham | 304 | 13 | NA^a^ | NA^a^ | NA^a^ | chromogenic ISH |
| sham | 305 | 13 | NA^a^ | NA^a^ | NA^a^ | IHC, Fluorescent ISH |
| sham | 306 | 9 | NA^a^ | NA^a^ | NA^a^ | IHC, Fluorescent ISH |
| paralyzed | 340 | 10 | 10^b^ | 6, 6 | 0 | IHC |
| paralyzed | 343 | 9 | 9^b^ | 5, 5 | 0 | IHC, Fluorescent ISH |
| paralyzed | 345 | 10 | 10^b^ | 6, 6 | 0 | chromogenic ISH |
| paralyzed | 347 | 10 | 10^b^ | 5, 6 | 0 | chromogenic ISH |
| paralyzed | 362 | 10 | 10^b^ | 6, 6 | 0 | chromogenic ISH |
| paralyzed | 338^d^ | 13 | 13^b^ | 6, 6 | 0 |  |
| paralyzed | 362 | 10 | 10^b^ | 6, 6 | 0 |  |
| paralyzed | 363 | 13 | 13^b^ | 6, 6 | 0 | IHC, Fluorescent ISH |
| paralyzed | 367 | 10 | 10^b^ | 5, 6 | 0 | IHC, Chromogenic ISH |
| paralyzed | 393 | 10 | 10^b^ | 6, 6 | 0 | IHC |
| paralyzed | 7 | 9 | 9^b^ | 6, 6 | 0 | IHC, Fluorescent ISH |
| days^c^ | 320 | 14 | 12 | 5, 3 | 2 | IHC |
| days^c^ | 337 | 13 | 10 | 6, 5 | 3 | IHC, Fluorescent ISH |
| days^c^ | 376 | 14 | 12 | 5, 6 | 2 | IHC, Fluorescent ISH |
| days^c^ | 395 | 14 | 12 | 5, 5 | 2 | IHC, Fluorescent ISH |
| 1 month^c^ | 350 | 41 | 13 | 6, 2 | 28 | IHC |
| 1 month^c^ | 383 | 41 | 12 | 5, 3 | 29 | IHC |
| 1 month^c^ | 387 | 41 | 11 | 2, 5 | 30 | IHC |
| 1 month^c^ | 391 | 41 | 15^f^ | 6, 4 | 26 | IHC |
| 2 months^c^ | 321 | 79 | 10^e^ | 3, 3 | 69 | IHC |
| 2 months^c^ | 348 | 79 | 12 | 4, 5 | 67 | IHC |
| 2 months^c^ | 390 | 79 | 10 | 6, 3 | 69 | IHC |
| 2 months^c^ | 11 | 79 | 15^f^ | 6, 4 | 56 | IHC |
| ^a^ Not applicable  ^b^ VPS not taken the day of sacrifice when the mice were paralyzed on both limbs (VPS equal to 5 or 6).  ^c^ Approximate time after paralysis that necropsy was performed.  ^d^ Freshly dead, was not going to recover. Rare animals in this model progress to severe disease and die.  ^e^ Highest VPS was 3.  ^f^ Stopped reading at day 15 until resumption of reading at day 37 when fully recovered (VPS equal to 0 or 1).  ISH – fluorescent *in situ* hybridization  IHC - immunohistochemistry | | | | | | |

**ImageJ protocols**

**IHC ChAT and ZIKV analysis (NIAW-984, Sidhu, 2023)**

1. Open tif file in imageJ
2. Image:color:make composite
3. Open color channels tool. Image:color:channels tool
4. Select color in channels tool.
5. Adjust brightness. Image:adjust:brighteness
6. Select area and mean grey scale as measurements. Analyze:set measurements.
7. Measure ChAT+ for each cell
   1. Click on Wand Tool.
   2. Double click on wand tool. Select outline of cell using Wand Tool window.
   3. Add each cell to ROI manager. Analyze:tools:ROI manager.
8. Select all ChAT+ cells in ROI manager. Click measure.
9. Copy measurements and paste in data spreadsheet.
10. Clear data from Results window.
11. Select channel 2 (ZIKV) in Channels Tool.
12. Measure
13. Copy and paste ZIKV data into spreadsheet.

**Colocalization image creator and analysis (publication)**

1. Load files into Fiji.
2. Convert files to 8-bit color
3. Image:color:merge
4. Image:color:channel tool. Select grey scale in menu bar of channel tool.
5. Plugins:colocalization image creator
6. Yes in Enable hotkeys window
7. Add element in colocalization image creator window
8. DID NOT DO THIS FOR FISH

**Analyze motor neurons in ventrolateral horn, estimated lamina IX** (NIAW-984)

1. Load individual tif images of ZIKV, ChAT and Syn into ImageJ.
2. Images:stacks:images to stack (check keep source images & use titles as labels)
3. Images:color:merge channels.
4. Increase intensity of ChAT channel
5. Analyze:Tool:ROI manager.
6. Select lamina IX
7. In ROI Manager, push Add[t]
8. Using wand, select ChAT+ neurons and Add[t] for each
9. Command:measure. Be sure that the correct window, channel are selected.

**ChAT RNA intensity in ventrolateral horn, estimated lamina IX** (NIAW-984)

1. Load individual tif images of ZIKV, ChAT and Syn into ImageJ.
2. Images:stacks:images to stack (check keep source images & use titles as labels)
3. Analyze:set measurements. Select Mean gray value.
4. Using image:adjust:brightness/contrast, outline the ventrolateral motor lobe using “free hand selection”
5. “reset” in B&C window
6. Measure ChAT intensity using command M
7. Move to ZIKV window using the menu bar at bottom of window
8. Process:math:subtract. Type 10 (background of negative sample).
9. OK, NO (Process Stack?)
10. Measure ZIKV intensity using command M
11. Move to SYN window using the menu bar at bottom of window
12. Measure SYN intensity using command M

**Make counted image for reference** (NIAW-776b)

1. Double-click on multi-count to increase size to very large.
2. Count motor neurons.
3. Make screen shot.
4. Rename (e.g. 102L1 count).
5. Save in Count folder.
6. If needed, we can get counts from this.

**Synaptophysin quantification in ChAT selected area** (NIAW-776b)

1. Make pdf screen shot of counted cells (above).
2. Open reference and original
3. Channel Color (not composite)
4. Sequentially from screen shot of counted cells, count #1, #2… etc):
   1. Double-click wand
   2. Adjust tolerance so that ChAT area is selected on channel 2. (Legacy)
   3. Run macro to increase size by 2 µm (channel 2).
      1. Experiment folder:analysis:enlarge selection macro.ijm
      2. Click on macro window
      3. Command R
      4. Notes: Edit:selection:enlarge (enter 2 µm)
   4. Measure color scale on channel 3

**ChAT mean gray value & count** (NIAW-947)

1. Count ChAT
   1. Open image in imageJ
   2. Adjust Brightness
   3. Count ChAT using Multi-point tool
   4. Shift command 4 to capture image
   5. Rename captured image
   6. Store in NIAW-947 ChAT count images
2. Mean gray scale
   1. Analyze:set measurements to Area and Mean gray scale
   2. Adjust Brightness
   3. Select ChAT using wand tool in numerical order using the desktop count image
   4. Command M
   5. Continue with all neurons in image
3. Spreadsheet – copy data to spreadsheet

**Intensity of NeuN in ChAT+ cells for figure** (NIAW-947)

1. Open multi-channel composite image
2. Open image:adjust:brightness/contrast
3. Open image:color:channels tool
4. Switch from Composite to Grayscale in channels tool
5. Increase brightness to see ChAT
6. Select nucleus two methods
   1. Option 1: On NeuN channel, select the obvious NeuN nucleus using the Wand tool
   2. Option 2:
      1. On the ChAT channel using Wand tool with tolerance set to 8 (double-click Wand tool), click center of neuron where nucleus is located.
      2. On the NeuN channel, enlarge the selection to encompass the area of interest using Edit:selection:enlarge
7. Command M to measure mean gray value

**merge ChAT NeuN for figure** (NIAW-947)

1. Open ChAT file and NeuN file in ImageJ
2. Image:adjust:brightness – adjust Max to ~150
3. Save tif of each.
4. Image:color:merge – make composite
5. More: convert to RGB
6. Save tif of merge

**Electron Microscopy**

**Prepare ahead of time:**

**1 L of 0.2 M Phosphate buffer (0.2 M PB), pH 7.4**

NaH2PO4-2H2O (monobasic, MW 155.99): 5.93 g

Na2HPO4 (dibasic, MW 141.96): 23.0 g

dH2O to 1 L

check pH = 7.4 (adjust with NaOH if necessary, NOT HCl – don’t want to make salt)

**1 L of 0.2 M Phosphate buffer (0.2 M PB), pH 7.4**

NaH2PO4-2H2O (monobasic, MW 155.99): 2.97 g

Na2HPO4 (dibasic, MW 141.96): 11.5 g

dH2O to 1 L

check pH = 7.4 (adjust with NaOH if necessary, NOT HCl – don’t want to make salt)

**500 ml of 0.02% CaCl2**

CaCl2-2H2O (147.02) 100 mg

dH2O to 500 ml

**EM fixative: 1% PFA, 2.5% GA**

| **ingredient** | **for 50 ml** | **for 200 ml** | **for 300 ml** | **for 500 ml** |
| --- | --- | --- | --- | --- |
| dH2O | 10 ml | 40 ml | 60 ml | 100 ml |
| 16% PFA(from vials) | 3.125 ml | 12.5 ml | 18.75 ml | 31.25 ml |
| 25% GA (EM grade) | 5 ml | 20 ml | 30 ml | 50 ml |
| 0.2 M PB | 25 ml | 100 ml | 150 ml | 250 ml |
| sucrose | 1.5 g | 6 g | 9 g | 15 g |
| 0.02% CaCl2-2H2O | 3 ml | 12 ml | 18 ml | 30 ml |
| dH20 | to final vol | to final vol |  |  |
| check pH = 7.4 |  |  |  |  |

*GA = glutaraldehyde

8/9/17

made 300 ml fresh EM fixative, store 4 deg

picked 1C-8 and 2A-10 for perfusion

**perfuse:** PBS to flush out blood, then fresh, room temperature EM fixative

**dissect out tissue:** vertebral column from about T8 to about S1 (to include lumbar spinal cord + landmarks for identifying specific levels)

**post-fix:** put dissected tissue in cold EM fixative, keep on ice until all tissue is collected, then post-fix, overnight, at 4 deg, on a rocker if possible

8/15/17

made 300 ml fresh EM fixative, store 4 deg

perfused 2A-11, 1A-15 and 1A-16 (perfusion on 1A-16 wasn’t as good as rest)

**perfuse:** PBS to flush out blood, then fresh, room temperature EM fixative

**dissect out tissue:** vertebral column from about T8 to about S1 (to include lumbar spinal cord + landmarks for identifying specific levels)

**post-fix:** put dissected tissue in cold EM fixative, keep on ice until all tissue is collected, then post-fix, overnight, at 4 deg, on a rocker if possible

**Day 2**

**rinse:** 0.1 M PB, 2X, 10 min

**dissect out L4-5 spinal cord:** remove the spinal cord from the vertebral column, keep a 2-3 mm thick section of the largest part (lumbar enlargement)

[anatomy notes: T12=last with ribs attached (ribs are attached at rostral edge of body), T9=angled spinous process, T10=stubby spinous process, T11=1^st^ with square spinous process, L4-5 spinal levels should be under T13 spinous process; can also find L4-5 spinal nerves and trace back to spinal cord (spinal nerves exit column on caudal side of body)]

**If shipping to UofU:** transfer tissue back into EM fixative (?) in small tubes

**If proceding in-house:** trim so tissue is < 1 mm thick & transfer to glass vials & cover with 0.1 M PB to keep moist & move to OsO4 hood (use only glass vials, etc – no plastic – and put all waste in glass waste container)

**Reagents:**

- 2% OsO4 in deionized water protected from light.
- Rinse a teflon-stoppered 100ml volumetric flask 3x with 0.1 N HNO3 and 3x with dH2O.
- Add 50 ml dH2O,
- Wash a 1 gram vial of OsO4 in warm soapy water.
- Wear goggles, apron, nitrile gloves and lay out paper towels in the hood.
- Place the vial in a beaker with hot tap water until the OsO4 crystals melt.
- Score the vial neck with a small trangular file, grasp wrapped in paper towels, break and drop into the volumetric flask.
- Sonicate until dissolved (several hours)
- Store wrapped in foil in hood.
- pH 5.15 Maleate buffer: 11.6 g maleate/500 ml dH2O, adjusted to pH 5.15 with NaOH
- pH 6.00 Maleate buffer: 11.6 g maleate/500 ml dH2O, adjusted to pH 6.00 with NaOH
- 1% uranyl acetate: dissolve in pH 6.00 maleate (pH drops on addition of uranyl acetate; dissolve by sonication)
- Procedure
- prepare fresh 1% OsO4: 1 ml 2% OsO4 stock + 1 ml 0.2 M PB in glass vial (with what?)
- remove PB, add 1% OsO4 to tissue so it just covers it
- stain with 1% OsO4 for 1 hr at RT
- rinse: 0.1 M PB, 2X, 10 min – put waste in OsO4 waste container
- rinse: Maleate buffer, pH 5.15, 5x, 15 min – put waste in OsO4 container?
- stain with 1% uranyl acetate for 1 hr at RT (wrap in foil)
- rinse: Maleate buffer, pH 5.15, 3x, 5 min – put waste in OsO4 container?
- Dehydration and Infiltration Schedule: All volumes 5-10 ml unless noted otherwise. This procedure is designed for tissue samples < 1 mm thickness, presumed to be in fixative.
- 10 m PB
- 10 m 75% MeOH
- 10 m 85% MeOH
- 10 m 95% MeOH
- 10 m 100% MeOH
- 10 m 100% MeOH
- 10 m 100% Acetone
- 10 m 100% Acetone
- 60 m 50% resin in acetone
- 4-20 h 75% resin in acetone
- 60 m 100% resin
- Mixing Active Resin: Mix resin fresh or thaw from freezer. Work quickly to prevent components from hydrating. Wear gloves as these components may cause contact dermatitis and the synthesis of certain components involves potential carcinogens. Prepare under a hood.
- Mix 2 volumes DDSA and 1 volume Medcast gently by hand with a spatula until the solution is clear (2-5 minutes)
- Add 0.6 ml DMP30 per 30 ml resin; Mix gently as before until clear (2-5 minutes). Solution will darken as the activated epoxide begins to bind oxygen.
- Use immediately or store in 30-60 cc syringe in the freezer. Expel all air before freezing and plug syringe with a wooden dowel. Do not cap with a needle or plastic cover as they will leak air.
- Embed samples in fresh resin in molds (position for cross section) and cure at 60-65 deg C, ON
- Section 70-80 nm
- Place sections on formvar grid
- Stain sections with uranyl acetate (5%, 1 hr?) and lead citrate (1%, 25 min?)
- Image on TEM
